# Predicting suicidal ideation in academic communities using machine learning methods: a cross-sectional study

**DOI:** 10.1016/j.lana.2026.101487

**Published:** 2026-04-30

**Authors:** Orlando Fernandes, Liana Catarina Lima Portugal, Priscila Maria de Oliveira da Fonseca, Débora Christina Muchaluat-Saade, Isabel Antunes David, Arthur Viana Machado, Eliane Volchan, Fátima Cristina Smith Erthal, Mirtes Garcia Pereira, Letícia de Oliveira

**Affiliations:** a Laboratório de Neurofisiologia do Comportamento, Departamento de Fisiologia e Farmacologia, Instituto Biomédico, Universidade Federal Fluminense, Rua Hernani Pires de Mello, 101, Niterói, Brazil; b Laboratório de Neurofisiologia, Departamento de Ciências Fisiológicas, Instituto de Biologia Roberto Alcantara Gomes, Centro Biomédico, Universidade do Estado do Rio de Janeiro, Boulevard 28 de Setembro, 87, Vila Isabel, Rio de Janeiro, RJ, Brazil; c Laboratório MídiaCom, Instituto de Computação, Universidade Federal Fluminense, Rua Passo da Pátria, 156, Niterói, RJ, Brazil; d Laboratório de Neurobiologia, Instituto de Biofísica Carlos Chagas Filho, Universidade Federal do Rio de Janeiro, Avenida Carlos Chagas Filho, 373, Rio de Janeiro, RJ, Brazil; e Laboratório Integrado de Pesquisa do Estresse (LINPES), Instituto de Psiquiatria, Universidade Federal do Rio de Janeiro, Avenida Venceslau Brás, 71, Rio de Janeiro, Brazil

**Keywords:** Suicidal ideation, Academic community, Machine learning, Depression, Loneliness, Childhood emotional maltreatment, Optimism

## Abstract

**Background:**

Research consistently shows that depression and suicidal ideation (SI) often cooccur. However, SI can arise without elevated depressive symptoms, suggesting that additional factors may also contribute. This study investigated the protective and vulnerability factors associated with SI beyond depressive symptomatology in the academic community.

**Methods:**

We employed multiple kernel learning (MKL) to distinguish participants with SI from those without SI. MKL incorporates the contribution of each psychometric instrument and specific items to the predictive model. Data were drawn from a large-scale online survey of the Brazilian academic community (N = 3828; 67.6% women; 33.2% black; mean age 38.28 years; SD 12.89 [95% CI: 31.6–33.1]). The models incorporated measures of depressive symptoms, optimism, loneliness, childhood maltreatment, and demographic characteristics.

**Findings:**

The MKL model accurately distinguished individuals with and without SI, achieving a mean balanced accuracy of 77.61% (95% CI: 77.40–77.82) and an area under the curve (AUC) of 0.862 (95% CI: 0.860–0.864). While depressive symptoms were strong predictors, other variables, such as optimism, loneliness, childhood emotional maltreatment, and demographic characteristics together accounted for half the total weight in the classification model.

**Interpretation:**

These findings underscore the need for suicidal ideation screening protocols that consider a broader range of emotional and behavioral factors beyond depressive symptoms, particularly within academic communities. These insights may inform the design of targeted interventions to promote mental well-being in academic settings.

**Funding:**

Carlos Chagas Filho Foundation of Research Support in Rio de Janeiro (FAPERJ: E-26.201.678/2022; E-26.201.118/2021).

## Resumo

**Contexto:**

Estudos mostram que a depressão e a ideação suicida (IS) frequentemente ocorrem em conjunto. No entanto, a ideação pode surgir mesmo sem níveis elevados de sintomas depressivos, sugerindo que outros fatores também podem contribuir para esse fenômeno. Este estudo investiga fatores de proteção e vulnerabilidade associados à ideação suicida, para além dos sintomas depressivos na comunidade acadêmica.

**Métodos:**

Foram aplicados métodos de aprendizado de múltiplos kernels (MKL) para distinguir indivíduos com ideação suicida daqueles sem ideação suicida. O MKL incorpora a contribuição de cada instrumento psicométrico e dos seus itens específicos para o modelo preditivo. Os dados foram obtidos a partir de uma pesquisa online em larga escala com a comunidade acadêmica brasileira (N = 3.828; 67.6% mulheres, 33.2% pessoas negras, idade média de 32.3 anos [IC 95%: 31.6–33.1]). Os modelos incluíram medidas de sintomas depressivos, otimismo, solidão, maus-tratos emocionais na infância e características demográficas.

**Resultados:**

O modelo de MKL distinguiu com precisão indivíduos com e sem ideação suicida, alcançando uma acurácia média de 77.61% (IC 95%: 77.40–77.82) e uma área sob a curva de 0.862 (IC 95%: 0.860, 0.864). Embora os sintomas depressivos tenham sido fortes preditores, outras variáveis, como otimismo, solidão, maus-tratos emocionais na infância e características demográficas, conjuntamente representaram cerca de metade dos pesos totais no modelo de classificação.

**Interpretação:**

Esses resultados destacam a necessidade de protocolos de rastreamento da ideação suicida que considerem um conjunto de fatores emocionais e comportamentais para além dos sintomas depressivos, especialmente em comunidades acadêmicas. Tais achados podem orientar o desenvolvimento de intervenções direcionadas à promoção do bem-estar mental em contextos acadêmicos.

**Financiamento:**

Fundação Carlos Chagas Filho de Amparo à Pesquisa do Estado do Rio de Janeiro (FAPERJ: E-26.201.678/2022; E-26.201.118/2021).

**Research in context**

**Evidence before this study**

We searched PubMed, Scopus, and PsycINFO through February 2026, and examined reference lists. Searches combined terms for suicidal ideation (SI), suicide risk, and suicide attempts; depressive symptoms (including PHQ-9); vulnerability and protective factors (e.g., optimism, loneliness, childhood maltreatment—including emotional abuse and neglect); and machine learning (including multiple kernel learning and other models), as well as regression methods and descriptive data. We considered observational studies focusing on academic populations that reported SI as an outcome or label in adults and adolescents. Although depression and SI frequently co-occur, the literature does not agree that SI arises only with increased depressive symptoms. Other factors—such as loneliness, comorbid psychiatric conditions, and childhood emotional maltreatment—may increase SI risk, whereas optimism may be protective. Prior machine-learning studies show good performance in predicting SI from diverse data sources, but interpretable models that quantify contributions at both the scale and item levels in academic settings remain limited. Common risks of bias include non-probabilistic sampling, self-report measures, and limited external validation.

**Added value of this study**

We used sparse multiple kernel learning to integrate psychometric and demographic data from a large academic community, achieving accurate classification of SI while quantifying contributions at both the instrument and item levels. Beyond depressive symptoms, loneliness, childhood emotional maltreatment, optimism, and demographics collectively accounted for approximately half of the model’s weight, enhancing interpretability. To address the class imbalance typical of SI datasets, we implemented repeated subsampling of the majority class with multiple resampled combinations and nested cross-validation, which mitigated imbalance and supported the reproducibility and robustness of the results.

**Implications of all the available evidence**

SI screening in academic settings should not rely solely on depressive symptoms. Multi-domain protocols that incorporate social connectedness, early emotional adversity, dispositional optimism, and demographics can improve risk stratification and guide targeted interventions.

## Introduction

Suicidal ideation refers to thoughts of wanting to die or intentionally ending one's own life and is a key indicator of suicide risk. Although its intensity and frequency may influence suicidal behavior,^1^ it is a multifaceted phenomenon shaped by both vulnerability and protective factors.^2^ Effective prevention depends on understanding these determinants, particularly in vulnerable populations such as the academic community. A meta-analysis revealed that 22.3% of college students worldwide experience suicidal ideation and that 3.2% attempt suicide.^3^ Among Brazilian undergraduates, the pooled prevalence of suicidal behavior is 9.10%, alongside high rates of anxiety (37.75%) and depression (28.51%).^4^ Other academic groups including PhD students (2–12%),^5^^,^^6^ and tenured university hospital faculty (15%), also have an elevated risk.^7^ Studies examining suicidal ideation across diverse populations in South America remain scarce, underscoring the need to identify vulnerability and protective factors to inform prevention strategies.

Depression is strongly associated with suicidal ideation and poses a major challenge to mental health in academic populations,^8^ particularly among undergraduate students.^9^ A meta-analysis revealed that individuals with depression are five times more likely to experience suicidal ideation than those without depression.^10^ This association is well established, with greater vulnerability observed among men, sexual minorities, multiracial young people, and individuals with comorbid psychopathologies.^11^ However, suicidal ideation can also occur with mild depressive symptoms, especially in the presence of additional vulnerabilities such as low social support and affective dysregulation.^12^ Additionally, other factors have also been implicated in academic settings among graduate and undergraduate students.^13^, ^14^, ^15^

Accordingly, we conceptualized suicidal ideation as a multifactorial outcome, integrating depressive symptoms with selected vulnerability and protective domains. We examined loneliness and childhood maltreatment as vulnerability factors, and optimism as a protective factor. These domains are highly relevant in academic populations. Loneliness has been identified as the only significant factor associated with suicidal ideation during the COVID-19 outbreak^16^ and remains a consistent risk factor among students, particularly alongside depression.^17^ Childhood maltreatment, especially emotional abuse, is also associated with higher suicidal ideation rates among students.^18^ In contrast, optimism appears to mitigate suicidal ideation beyond the effects of depression^19^ and may serve as a protective factor against suicidal ideation development by transcending depressive symptomatology.^20^ Together, these factors likely contribute in complementary ways to suicidal ideation, supporting the integration of vulnerability and protective mechanisms in predictive models.

Machine learning methods have been used as effective screening and evaluation tools for identifying vulnerability and protective factors for suicidal ideation.^13^^,^^15^ They detect multivariate patterns in complex psychometric data and enable robust individual-level classification.^21^ Systematic reviews show that good performance can be achieved with few features, although generalization to independent samples remains a key challenge.^21^ Nevertheless, machine learning offers advantages, including fewer assumptions, multivariate modeling, and suitability for complex datasets.^22^

Studies have applied machine learning to detect suicidal ideation from neuroimaging data,^23^ social media data,^24^ clinical interviews, and medical records.^15^ More recently, these models have also been shown to be effective when self-report scales are used among undergraduate students.^17^ A range of algorithms have been employed, including support vector machines (SVMs), logistic regression, decision trees, random forests, and artificial neural networks.^13^^,^^17^ While linear models, such as linear SVM and logistic regression, offer direct insights into the contribution of each variable to predictions, nonlinear models, such as artificial neural networks, require post hoc explanatory techniques. However, even in linear multivariate settings, identifying the most relevant predictors among correlated variables remains challenging.

Sparse methods, such as least absolute shrinkage and selection operator (LASSO)^25^ or elastic net,^26^ improve interpretability by decreasing coefficients to zero and selecting relevant variables. These approaches can operate at the variable or group level, with group sparsity enabling the selection of related blocks, such as questionnaire subscales. Multiple kernel learning (MKL) extends this framework by integrating heterogeneous data sources.^27^ Sparse MKL can identify informative data types or features subsets and has been applied in neuroimaging to capture whole-brain and regional contributions.^28^, ^29^, ^30^ When applied to psychometric data, MKL enables integration across instruments while estimating contributions at both the scale and the item level, preserving interpretability and helping identify key predictors relevant for intervention.

This study aimed to examine psychosocial vulnerability and protective factors associated with suicidal ideation among an academic population, beyond depressive symptoms. By jointly considering domains such as loneliness, optimism, childhood emotional maltreatment, and demographic characteristics, we sought to provide a more comprehensive understanding of suicidal ideation and its potential implications for early identification and prevention.

## Methods

A detailed version of all the methods is provided in the Supplementary Methods.

### Study design and participants

**Fig. 1:**
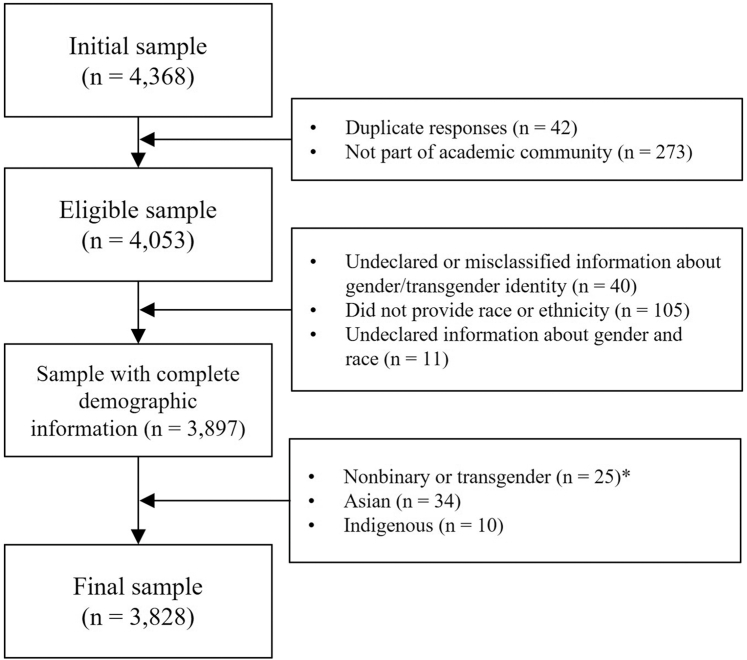
**Flowchart of the sample inclusion and exclusion criteria.** A total of 4326 responses were collected. The final analytic sample included 3828 respondents. ∗Note: A “prefer not to answer” option was available for the sociodemographic items; these, along with ambiguous open-text responses, were categorized as undeclared responses and excluded from the main models. Detailed information for the gender, race, or ethnicity identity subgroups is presented in Supplementary Table S2.

This study is part of the PSIcovidA project, a longitudinal study conducted in Brazil that focused on mental health in the academic community, with data collected to assess multiple psychosocial domains. Data were collected using an anonymous web-based survey implemented on the Google Forms platform, and the procedures are reported in accordance with the Checklist for Reporting Results of internet E-Surveys (CHERRIES).^31^ The survey was conducted during the return to in-person activities after the COVID-19 pandemic, from March 10 to June 10, 2022. Participants were recruited via convenience and snowball sampling^32^ through institutional and personal email lists, with questionnaires distributed through email, online messaging platforms (e.g., WhatsApp), and social media (@projetopsicovida). This study was approved by the Ethics Research Committee of Fluminense Federal University (UFF) and the National Research Ethics Commission (CAAE: 52739721.0.0000.5243). Participants provided electronic informed consent prior to participation; those who declined were redirected to a mental health information page, and participants could withdraw at any time. Participants completed demographic and psychometric questionnaires, which took approximately 25 min. Upon completion, participants received mental health guidance and contact information for psychological support. A total of 4326 responses were collected through a web-based survey of academic faculty, undergraduate and graduate students, postdoctoral researchers, and administrative staff at universities and research institutes. The exclusion criteria included duplicate responses (N = 42), nonacademic responses (N = 273), undeclared or misclassified gender identity (N = 40), undeclared race or ethnicity (N = 105), and undeclared information for both gender and race/ethnicity (N = 11). Owing to small subgroup sizes, nonbinary and transgender individuals (N = 25), Asian individuals (N = 34), and Indigenous individuals (N = 10) were excluded from the classification models, as reliable statistical estimation was not feasible. Detailed information for gender identity and race or ethnicity subgroups is presented in Supplementary Table S2. The final sample consisted of 3828 respondents (Fig. 1). Table 1 provides detailed demographic information.

**Table 1:** Demographic and psychometric characteristics of the sample.

	Total sample	With SI	Without SI	χ^2^	df	*p* values
	N (%)	N (%)	N (%)			
	3828 (100)	722 (100)	3106 (100)			
Gender						
Men	1239 (32.37)	195 (27.01)	1044 (33.61)	11.67	1	0.0006
Women	2589 (67.63)	527 (72.99)	2062 (66.39)			
Race or ethnicity						
White individuals	2555 (66.75)	413 (57.20)	2142 (68.96)	36.51	1	<0.0001
Black individuals	1273 (33.25)	309 (42.80)	964 (31.04)			

	Mean (95% CI: lower; upper)			χ^2^	df	*p* values
	38.20 (37.79–38.60)	32.34 (31.56–33.13)	39.56 (39.11–40.01)			
	N (%)	N (%)	N (%)			
Age						
18–29	1128 (29.47)	348 (48.20)	780 (25.11)	183.23	4	<0.0001
30–39	1089 (28.45)	195 (27.01)	894 (28.78)			
40–49	819 (21.39)	113 (15.65)	706 (22.73)			
50–59	525 (13.71)	56 (7.76)	469 (15.10)			
60 or more	267 (6.97)	10 (1.39)	257 (8.27)			
Previous mental disorders						
Yes	1361 (35.55)	400 (55.40)	961 (30.94)	152.99	1	<0.0001
No	2467 (64.45)	322 (44.60)	2145 (69.06)			

Psychometric measures						
	Mean (95% CI: lower-upper)			T value	df	*p* values
TILS	5.33 (5.28–5.39)	6.70 (6.58–6.83)	5.02 (4.96–5.08)	−23.51	3826	<0.0001
CTQ-EA	10.11 (9.96–10.25)	13.27 (12.90–13.64)	9.37 (9.23–9.52)	−21.82	3826	<0.0001
CTQ-EN	11.20 (11.06–11.35)	13.98 (13.63–14.33)	10.56 (10.40–10.71)	−18.79	3826	<0.0001
LOT-R	15.03 (14.86–15.20)	10.82 (10.43–11.22)	16.01 (15.83–16.18)	24.85	3826	<0.0001
PHQ^a^	8.75 (8.53–8.96)	15.60 (15.17–16.02)	7.16 (6.95–7.36)	−34.70	3826	<0.0001

Data are presented as N (%), where percentages represent the proportion within each group and sum to 100% within each column. The columns represent the total sample (all participants), with SI (participants with suicidal ideation), and without SI (participants without suicidal ideation).

a PHQ—assessed by the sum of items 1–8 depressive symptoms, excluding item 9. SI: suicidal ideation; TILS: loneliness; EA: emotional abuse (CTQ-CM); EN: emotional neglect (CTQ-CM); LOT-R: Life Orientation Test-Revised; PHQ: depressive symptoms; *df*: degrees of freedom; SD: standard deviation; CI: confidence intervals.

### Outcome (label): assessment of suicidal ideation

Suicidal ideation was assessed using Item 9 of the Patient Health Questionnaire (PHQ-9).^33^^,^^34^ This item evaluates passive thoughts of death or self-injury and is often used to screen depressed patients for suicide risk.^34^^,^^35^ Participants answered the following question: “How often have you been bothered by thoughts that you would be better off dead or of hurting yourself in some way?” The responses included “not at all” (0), “several days” (1), “more than half the days” (2), and “nearly every day” (3) over the past 2 weeks. For the classification models, a positive suicide risk screen indicated any nonzero response (score ≥1), whereas a score of 0 indicated negative screening. This measure has been widely used in studies investigating the risk for suicidal ideation,^36^ including in academic populations.^37^

### Psychometric variables included in the predicted models

Demographic information included gender (“women”/“men”), age, race or ethnicity, and history of mental disorders. All gender and demographic information was self-reported by participants through predefined response options. Owing to the small subgroup sizes, reported genders other than men and women were not included in the classification models. Terminology for race or ethnicity followed the *Brazilian Institute of Geography and Statistics* (IBGE) census categories—White, Black, Brown, Asian, and Indigenous. Throughout the report, the black category encompasses both black and brown individuals. A history of mental health disorders was assessed via self-report. Participants were asked whether they had ever received a diagnosis of a mental disorder from a health professional (including depression, anxiety disorders, bipolar disorder, or substance use disorders). Demographic information was encoded using one-hot representations for categorical variables, including gender, race and the presence of previous mental disorders. Age was treated as a continuous value.

Depressive symptom severity was modeled as a continuous value and assessed using the PHQ-9 items 1–8,^33^^,^^34^ while item 9 (suicidal ideation) was used as the outcome. Loneliness was assessed by the Three-Item Loneliness Scale (TILS).^38^^,^^39^ Emotional abuse and emotional neglect were assessed with the Portuguese adapted version of the Childhood Trauma Questionnaire (CTQ),^40^^,^^41^ which includes five items for each subtype of childhood maltreatment (physical, emotional, and sexual abuse) as well as physical and emotional neglect. Only the emotional subtypes of childhood maltreatment (emotional abuse and emotional neglect) were used. Optimism was assessed by the Life Orientation Test-Revised (LOT-R), a questionnaire designed to assess the way individuals perceive their lives, either in a more or a less optimistic manner.^42^^,^^43^ Detailed descriptions of the psychometric scales are provided in the Supplementary Methods (study design and psychometric assessments).

### Data analysis—machine-learning classification models

Descriptive and inferential statistical analyses were conducted using JASP (version 0.18.3), while pattern classification analyses were performed in the Pattern Recognition for Neuroimaging Toolbox (PRoNTo, version 3).^44^ The dataset consisted of patterns of psychometric and sociodemographic variables along with labels indicating individuals with and without suicidal ideation. Psychometric data were treated as continuous, with each questionnaire item as one feature. One kernel matrix was created for each psychometric scale, and an additional kernel matrix was constructed from demographic variables (gender, race or ethnicity, age, and history of mental health disorders).

We used sparse multiple-kernel learning (SimpleMKL)^45^ as the primary classifier. The MKL simultaneously learns from and integrates multiple types of data, each represented by a separate kernel,^46^ while the weight of each kernel is estimated according to its contribution to prediction. Each psychometric variable was modeled as a separate kernel, enabling the MKL model to estimate both the overall contribution of each psychometric scale and demographic feature set (instrument-level contributions or kernel weights), as well as feature weights within each kernel (item-level contributions or item weights). This situation creates a hierarchical framework in which models derived from each psychometric variable are integrated into a global predictive model. For each variable, a linear kernel was computed on the basis of the item-level values. The MKL model in PRoNTo enforces sparsity through L1 regularization, ensuring that only a subset of kernels have a nonnull contribution to the predictive model (see the Supplementary Methods for a technical overview of the MKL algorithm). This approach enhances interpretability, as the model assigns a weight vector that represents both kernel-level and item-level contributions. In addition to the MKL algorithm, support vector machines^47^ and L2-regularized logistic regression^48^ were implemented to test the robustness of the classification models.

**Fig. 2:**
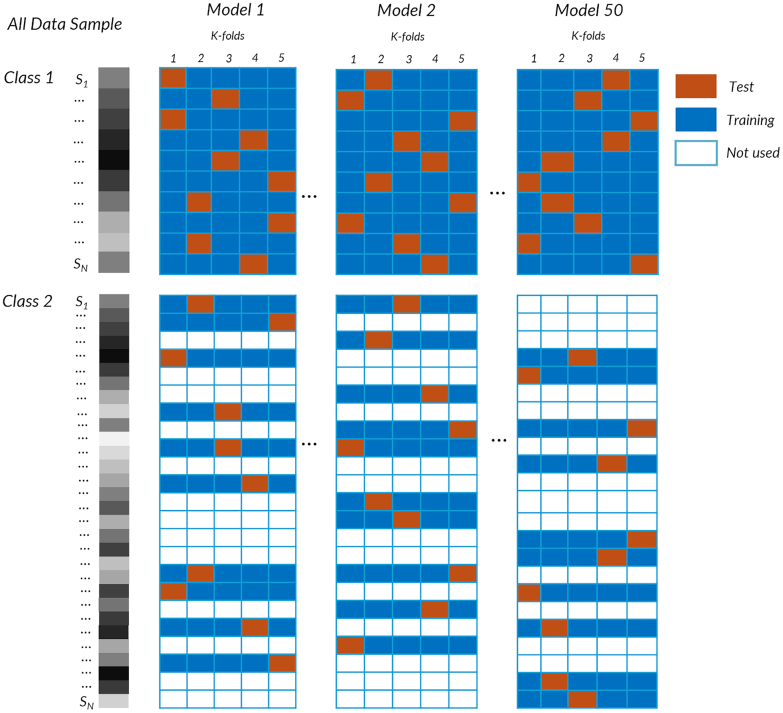
**Resampling methods for participant classification.** Participants with and without suicidal ideation (SI) were classified on the basis of their psychometric and demographic data. In the left panel, the full sample is divided into the SI group (Class 1: N = 722) and the without SI group (Class 2: N = 3106), with each row representing one participant (S_1_ to S_n_). The right panel illustrates the random undersampling strategy. Models 1–50 represent repeated runs in which the SI group remains fixed, while different participant subsets are drawn randomly from the without-SI group to create balanced datasets. The colored squares illustrate the k-fold cross-validation procedure (k = 5), where the SI group is randomly combined with different resampled subsets from the without-SI group to create balanced training and testing datasets. This procedure allows for comprehensive undersampling across the participants, allowing the model to achieve stable estimates and interpretable contributions to SI classification.

To address class imbalance (with suicidal ideation: N = 722; and without: N = 3106), we repeatedly undersampled the majority class. For each iteration, we randomly selected 722 individuals without suicidal ideation to match them with the other group and then performed nested 5-fold cross-validation on the new match set. This process, random selection, fold assignment, training, and testing, was repeated fifty (50) times (Fig. 2 provides a visual representation of the procedure). We report the means and confidence intervals (95% CIs, lower and upper) for the performance values. For each model, the cross-validation consisted of nested cross-validation with k = 5 for both the internal and external loops. An internal loop was used to optimize the models’ hyperparameters, and an external loop was used to assess the models’ performance. All preprocessing steps, including mean-centering, standardization, and kernel normalization, were performed within each training fold and subsequently applied to the corresponding test fold to avoid information leakage. For nonkernel models (L2-logistic regression), psychometric features were *z scored* within each training fold and applied to the test fold. As detailed in the Supplementary Materials (in the section on machine learning classification models), we further evaluated an alternative oversampling strategy using the synthetic minority oversampling technique (SMOTE) to assess the stability of the findings reported with random undersampling in the fifty models. The convergence of results across undersampling and oversampling approaches provides additional evidence for the consistency and robustness of the findings. The performance was assessed using balanced accuracy, class specific accuracy, the area under the curve (AUC), precision, recall, specificity and F1 scores (these methods are described in detail in the Supplementary Methods, section Performance Metrics).

The statistical significance of the models’ performance was assessed using a nonparametric permutation test with a significance threshold of *p*< 0.05. Since the analytical framework involved 50 separate undersampled datasets, the permutation procedure was conducted independently for each of the 50 models. For each individual model, the entire training and testing procedure was repeated 100 times with randomly permuted labels to generate a null distribution.^9^ The *p* value was calculated as the proportion of permutations in which the performance metric with permuted labels was equal to or greater than the metric obtained with the true labels (or equal to or smaller, in the case of the AUC). Model performance was considered statistically significant when the true metric results exceeded the permuted distribution in fewer than 5% of the iterations.

The model interpretation considered contributions at both the scale level (kernel weights) and the item level (item weights) to the linear predictive function. The MKL approach produces the following two sets of weights: kernel weights, reflecting the overall contribution of each psychometric scale, and item weights, representing the influence of each specific question. Positive weights indicate stronger contributions toward identifying individuals in the suicidal ideation group (higher risk of SI), whereas negative weights indicate stronger contributions toward identifying individuals in the group without SI (lower risk of SI). The kernel- and item-level contributions from the psychometric and demographic variables across the 50 classification models with random undersampling are summarized in the Results section, and the detailed results are provided in the Supplementary Materials, including the weights for the SVM and L2-logistic regression models.

### Role of the funding source

Funders played no role in the study design, data collection, analysis, interpretation, or writing.

## Results

### Sample characteristics

The final sample of 3828 individuals was used for further analysis, and Table 1 provides demographic information, including age, gender, race or ethnicity; the presence of previous mental disorders; and the values of psychometrics scales for these samples. The sample was mostly composed of women (N = 2589; 67.63%) and White individuals (N = 2555; 66.74%), aged between 18 and 29 (N = 1128; 29.47%) and 30–39 years (N = 1089; 28.49%).

Suicidal ideation was present in 18.86% (722/3828) of the academic community sample. As expected, Table 1 shows the differences in all the demographic characteristics between the groups with and without suicidal ideation. We found significant differences between the groups with and without suicidal ideation in terms of gender (χ^2^= 11.67, *p* = 0.0006) and between groups with a significant difference in terms of race or ethnicity (χ^2^= 36.51, *p*< 0.0001) and a previous history of mental disorders (χ^2^= 152.99, *p*< 0.0001). Compared with the other age groups, the group aged 18–29 years had the greatest prevalence of suicidal ideation (30.85%; 348/1128). Suicidal ideation was more prevalent among women, black individuals, young individuals, and individuals with a history of mental illness. Furthermore, all the psychometric scales differed between the groups with and without suicidal ideation. Specifically, the suicidal ideation groups presented greater levels of loneliness, depressive symptoms, and childhood emotional maltreatment scores for both subscales and lower optimism levels. In general, all aspects of mental health in the suicidal ideation group were more severe.

### Suicidal ideation risk identification

**Fig. 3:**
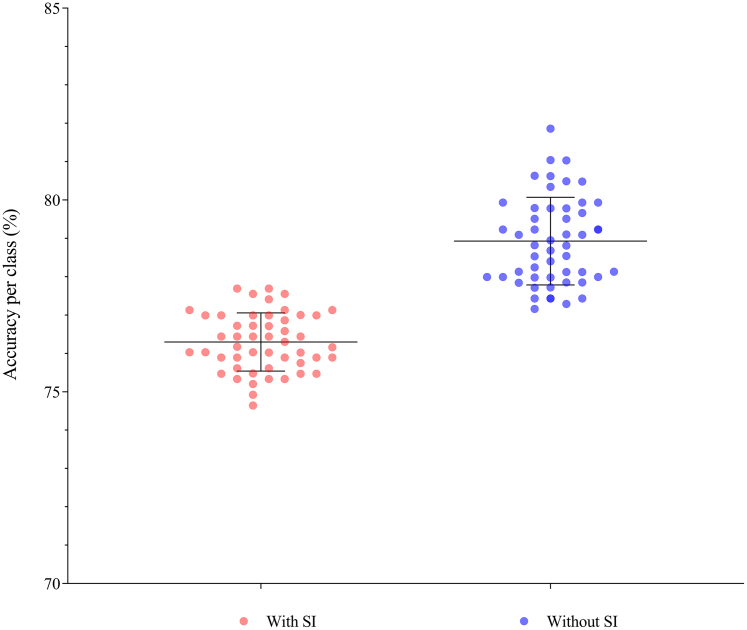
**MKL classification algorithm results.** In terms of accuracy per class, red dots represent individuals with suicidal ideation (SI) symptoms, and blue dots represent those without SI symptoms. Each dot represents a set of individuals from one of the 50 datasets randomly selected for each independent stage of the classification models. Lines represent the mean and standard deviation of the accuracy values.

The MKL classification models accurately distinguished between groups of individuals who had suicidal ideation and those who did not have such ideation. The average balanced accuracy for the fifty models was 77.61% (95% CI: 77.40–77.82), with a mean AUC of 0.86 (95% CI: 0.860–0.864). The average accuracy for discriminating the group with suicidal ideation was 76.30% (95% CI: 76.08–76.51), whereas for discriminating the group without suicidal ideation, it was 78.93% (95% CI: 78.60–79.25) (Fig. 3). Following the permutation test, all the models had *p values* lower than 0.05 for balance accuracy and accuracy for each class; for a detailed description, see the Supplementary Results (Supplementary Tables S3–S6). These results are also confirmed by other metrics, such as the precision, specificity, recall, and F1 scores presented in Table 2. The accuracy of the other algorithms (SVM and L2-logistic regression) was greater than 75%, and the AUC was greater than 0.85.

**Table 2:** Results for all algorithms and the average of fifty models with random groups.

Metrics	L1—MKL^a^	SVM^a^	L2-LR^a^
	Mean (95% CI: lower-upper)		
Balance accuracy (%)	77.61 (77.40–77.82)	78.23 (78.02–78.44)	78.57 (78.37–78.78)
Accuracy (SI group)	76.30 (76.08–76.51)	77.07 (76.79–77.34)	77.92 (77.69–78.14)
Accuracy (no-SI group)	78.93 (78.60–79.25)	79.39 (79.11–79.67)	79.23 (78.96–79.50)
AUC	0.862 (0.860–0.864)	0.856 (0.854–0.858)	0.871 (0.869–0.873)
Precision	0.784 (0.781–0.786)	0.789 (0.787–0.791)	0.790 (0.787–0.792)
Specificity	0.789 (0.786–0.792)	0.794 (0.791–0.797)	0.792 (0.790–0.795)
Recall	0.763 (0.761–0.765)	0.771 (0.768–0.774)	0.779 (0.777–0.782)
F1-score	0.773 (0.771–0.775)	0.780 (0.778–0.782)	0.784 (0.782–0.786)

SI, suicidal ideation; AUC, area under the curve of the receiver–operating characteristic; 95% CI, confidence interval of the mean; MKL, multiple kernel learning; SVM, support vector machine; L2-LR, L2-logistic regression.

a All fifty random undersampled models achieved statistical significance (*p*< 0.05) for balanced accuracy, class-specific accuracy, and AUC. *p values* were derived from nonparametric permutation tests (100 permutations per model). The asterisks indicate that 100% of the models performed significantly better than chance (AUC = 0.5 or accuracy = 0.5) across all individually performed permutation tests. The detailed results of the permutation testing are provided in the Supplementary Materials.

### Model interpretation—identifying predictors of suicidal ideation

**Fig. 4:**
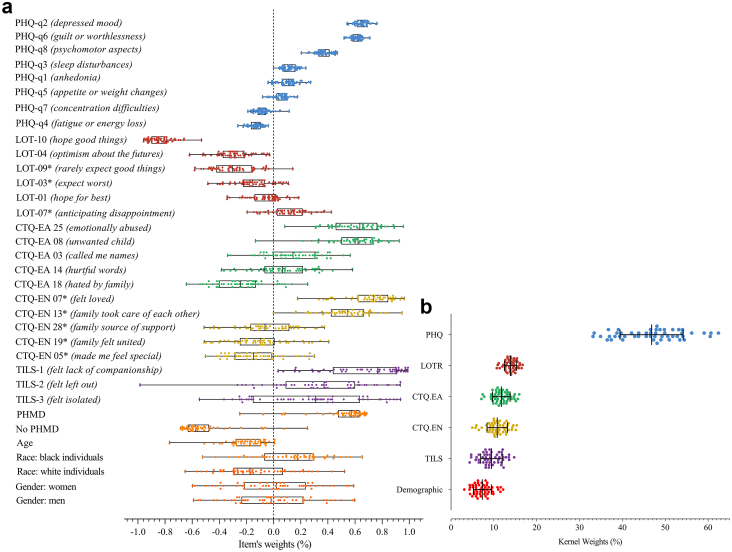
**Results of the item and kernel weights for the classification algorithm. (a)** Item-level weights for each psychometric scale and demographic variable. Each point represents the estimated weight from one of the fifty randomly undersampled models. The rectangular boxes indicate the mean and standard deviation across models, whereas the whiskers represent the minimum and maximum values. The vertical dashed line indicates zero, separating positive and negative weight contributions. **(b)** The kernel weights represent the contribution of each psychometric instrument to the predictive model. Each point corresponds to one undersampled model, and the boxplots summarize the distribution of the kernel weights across the fifty (50) models. The psychometric scales included the Patient Health Questionnaire (PHQ; depressive symptoms), the Life Orientation Test–Revised (LOT-R; optimism), the Childhood Trauma Questionnaire–Emotional Abuse (CTQ-EA), the Childhood Trauma Questionnaire–Emotional Neglect (CTQ-EN), the Three-Item Loneliness Scale (TILS), and demographic variables (age, gender, and race or ethnicity). ∗Items were reverse scored to ensure directional consistency within each construct, with higher scores uniformly representing greater construct severity (e.g., lower-level optimism or higher-level neglect).

As expected, depressive symptoms emerged as the strongest discriminator, accounting for a mean kernel weight of 46.83% (SD 7.37) across the fifty models. Within this scale, depressive mood and guilt had the highest item-level weights, indicating that greater levels of these symptoms increased the likelihood of suicidal ideation classification. Optimism was the second most influential factor (mean kernel weight of 13.89%, SD 1.41), with item-level results showing that higher optimism scores were associated with a lower suicidal ideation risk, which is consistent with its protective role. Childhood emotional abuse also contributed substantially, with emotional abuse (mean kernel weight of 11.74%, SD 2.04) and emotional neglect (mean kernel weight of 10.79%, SD 2.39) having similar effects. Items reflecting emotional mistreatment, lack of familial love, and perceived neglect were key contributors to suicidal ideation classification. Loneliness contributed moderately to the model (mean kernel weight of 9.43%, SD 2.51), with perceived lack of companionship emerging as its most salient feature, followed by social exclusion and isolation, which were associated with increased suicidal ideation risk. The demographic variables collectively accounted for an average of 7.31% of the kernel weights (SD 2.10). Among these, a history of mental illness was a stronger contributor to suicidal ideation classification, whereas its absence was associated with lower risk. The results are presented in Fig. 4 and Table 3, with additional details in the Supplementary Material. The feature weights from the SVM and L2-regularized logistic regression models showed similar patterns, supporting the consistency of the findings across the models (Supplementary Fig. S1 and Table S7).

**Table 3:** The kernel weight results for suicidal ideation classification for MKL models with fifty random group samples.

Predictors			95% CI of Mean		Range	
	Mean	SD	Lower	Upper	Min.	Max.
Depressive symptoms (PHQ-8 items)	46.83	7.37	44.73	48.92	33.14	62.42
Optimism (LOT-R)	13.89	1.42	13.49	14.30	10.68	16.72
Emotional Abuse (CTQ-CM)	11.74	2.04	11.16	12.32	7.10	15.92
Emotional Neglect (CTQ-CM)	10.79	2.39	10.11	11.47	4.97	15.35
Loneliness (TILS)	9.43	2.51	8.72	10.15	4.35	14.74
Demographic variables	7.31	2.10	6.72	7.91	3.10	12.16

SI: suicidal ideation; MKL: multiple-kernel learning; SD: standard deviation; CI: confidence interval; Min, minimum; Max, maximum.

## Discussion

Our findings demonstrate that MKL and other algorithms (SVM and L2-logistic regression) can effectively distinguish individuals with suicidal ideation from those without suicidal ideation. As expected, high levels of depressive symptoms were more strongly associated with classification as having suicidal ideation. However, other factors, such as optimism, loneliness, childhood emotional maltreatment (including abuse and neglect), and demographic information, accounted for approximately half of the overall weight, highlighting the importance of screening protocols that consider a range of emotional and behavioral factors beyond depressive symptoms in the academic community. Interestingly, optimism had a negative weight, indicating that individuals with high optimism scores were more likely to be classified without suicidal ideation, suggesting that optimism is an important protective factor against suicidal ideation. These findings may inform prevention strategies in higher education settings. These findings can be interpreted within the three-step theory of suicide, which posits that suicidal ideation arises from the joint presence of psychological pain and hopelessness.^49^ Accordingly, childhood emotional maltreatment and loneliness may be conceptualized as distinct contributors to psychological pain, both of which are independently associated with suicidal ideation beyond depression, whereas optimism has been identified as a protective factor against the effects of hopelessness.^49^^,^^50^

Within this prediction-focused framework, a key strength was the use of undersampling to address class imbalance by generating matched subsamples with equal class sizes and averaging performance. This is relevant because many algorithms favor the majority class, reducing accuracy under imbalance. Given the larger nonsuicidal ideation group, we created 50 matched samples via random undersampling and performed independent 5-fold cross-validation in each iteration. The results revealed stable model performance, indicating robustness, although the demographic weights varied more. This approach may enhance reproducibility in suicide research. Most studies in the academic community have focused on undergraduate students in high-income countries,^15^^,^^37^ although recent investigations have emerged from middle-income and developing countries.^8^^,^^13^^,^^14^ In this study, we investigated the risk and protective factors for suicidal ideation across all academic positions in a developing country (Brazil). This focus is particularly relevant given that a recent meta-analysis revealed suicidal behaviors in almost one in 10 Brazilian undergraduate students.^4^ Additionally, caution is warranted when extending these results are extended to academic communities in other sociocultural contexts.

As expected, our MKL classification models revealed that compared with the other variables, depressive symptoms contributed the most (46.85% kernel weights). These results align with many machine learning studies showing that depressive symptoms influence classification models, especially among university students.^13^^,^^14^^,^^17^ Furthermore, MKL enables the estimation of kernel weights that reflect the contribution of the psychometric scale as a whole and of each specific question. Here, we found that two specific depressive symptoms (depressed mood and guilt) had greater positive weights for predicting suicidal ideation. There is considerable evidence that guilt is strongly associated with suicidal ideation, including in clinical populations.^51^ In fact, guilt mediates the association between depressive symptoms and suicidal ideation in military samples.^52^ Similarly, depressive mood characterized by persistent sadness, emptiness, or hopelessness, in addition to anhedonia, was identified as a key predictor of suicidal ideation in a machine-learning study of students.^13^ In addition, increased suicidal ideation and depressive symptoms in academic populations during and after the COVID-19 pandemic may have contributed to these patterns.^53^ Collectively, these findings suggest that guilt and depressed mood, along with other depressive symptoms, are particularly informative indicators for identifying suicidal ideation across diverse samples, including academic populations.

Interestingly, the optimism scale was the second strongest psychometric contributor to the classifier (after depression symptoms), with most kernel item weights being negative. In this study, we conceptualized optimism as a trait reflecting how individuals appraise their lives (more or less hopeful). Notably, the item with the greatest negative weight was *“I hope more good things happen than bad* things”. Optimism has been identified as a protective factor against mental disorders in the academic community, especially among college students.^54^^,^^55^ Our findings align with evidence that optimism is linked to lower levels of suicidal ideation among college students.^56^^,^^57^ Optimism has also been identified in machine-learning studies as a significant factor for predicting suicide attempts at the one-year follow-up in a group of young adults.^15^ Identifying protective factors may inform therapeutic interventions aimed at reducing suicidal ideation in the academic population. Interventions targeting protective factors, such as positive experiences, optimism, hope, and creativity,^58^ have been shown to reduce depression and suicidal ideation while improving self-esteem and positive outlook.^59^

In the present study, childhood emotional maltreatment accounted for approximately 22% of the kernel weights (emotional abuse: 11.79%; emotional neglect: 10.79%). These findings align with evidence that adverse childhood experiences have lasting mental health effects.^60^^,^^61^ Emotional abuse was associated with increased revictimization and posttraumatic stress disorder (PTSD) symptom severity in a college student sample^60^ and had the strongest effect on suicidal ideation among university students.^18^ Similarly, childhood maltreatment, including emotional abuse and neglect, is associated with an increased risk of suicidal ideation and related behaviors.^62^ With respect to machine learning, prior work has shown that using machine learning approaches, maltreatment and psychological factors can predict suicidal ideation among preadolescents.^63^ Our model extends this literature by showing that items from the emotional abuse subscale, such as *“felt emotionally abused”* and *“felt to be an unwanted child”*, carried greater positive item weights. With respect to the emotional neglect subscale, these items were reverse scored so that greater values reflected lower perceived affection and care, indicating greater emotional neglect and increased suicidal ideation risk. Overall, emotional maltreatment predicts suicidal ideation beyond depression symptom severity.

Finally, self-perceived loneliness contributed moderately to the classifier, accounting for 9.43% of the total kernel weights. Prior studies have consistently associated loneliness and suicidal ideation across populations,^64^ including students^65^ and Brazilian samples.^16^ In the general population, this association remains significant after controlling for depression and prior mental disorders.^66^ Specifically, loneliness has both direct and indirect effects on suicidal ideation.^64^ In our data, the item *“felt lack of companionship”* had the greatest item weight, indicating an increased risk of suicidal ideation classification. Loneliness may increase the desire to reestablish social connections while heightening vulnerability and threat sensitivity.^67^ Thus, interventions that increase belongingness and reduce perceived burdensomeness may be important for reducing suicidal ideation.^68^ Public awareness campaigns may further support individuals experiencing loneliness.

With respect to demographics, these factors contribute less to the model, accounting for 7.31% of the kernel weights. Weights should be interpreted with caution, as all the variables contributed to the final prediction. Prior psychiatric diagnoses contributed more strongly than other demographic variables did, and previous studies have associated mental disorder history and comorbidities with a greater risk of suicidal ideation.^17^ As shown in Fig. 4 and Table 3, demographic effects varied across the models; only age showed a consistent negative association with suicidal ideation. Although suicidal ideation was more prevalent among women and black individuals, these variables contributed little to the models. This finding should not be interpreted as a lack of relevance of gender- and race-related differences but as a limitation of the modeling approach.

Although this study provides valuable insights, several limitations should be acknowledged. First, the cross-sectional design precludes causal inferences. Second, self-reported data may introduce social desirability and recall biases, and interrelated psychosocial constructs, particularly depressive symptoms, may influence responses; however, this situation reflects real-world conditions and was addressed using a multivariate predictive framework to assess relative contributions. Third, suicidal ideation was assessed using a single item, which may limit measurement reliability and does not capture the full severity or heterogeneity of suicidal thoughts. However, this decision was motivated by the study context. In the setting of an anonymous online survey administered to a broad academic population, the use of a brief screening item helped minimize individuals’ burden and potential discomfort associated with completing multi-item questionnaires assessing suicidal ideation. Single-item assessments, including the PHQ-9 Item 9, are widely used in large-scale epidemiological and clinical studies and have demonstrated utility for screening and risk stratification.^69^^,^^70^ Additionally, a history of prior suicide attempts was not included because the survey instrument did not assess this information. Future studies should incorporate validated multi-item instruments to improve measurement precision.^71^ Fourth, generalizability is limited by the use of an academic sample and data collection during the pandemic, which may have increased psychological symptoms and affected the applicability of the findings to other populations or periods. The exclusion of nonbinary and transgender individuals and some racial and ethnic groups because of their small sizes further limits generalizability and may overlook relevant differences in suicidal ideation. Although machine learning is often criticized for its limited interpretability, this was partially mitigated by the use of linear models allowing examination of scale and item contributions; however, translating these models into clinical or institutional practice remains a challenge.

The clinical and social relevance of this approach lies in the fact that asking about suicidal ideation at a single time point provides limited insight into the upstream processes that precede its emergence. Suicidal behavior is widely conceptualized as a continuum encompassing suicidal ideation, plans, attempts, and death by suicide,^72^ with strong evidence indicating that suicidal ideation is a key antecedent of later suicidal behavior.^73^ In contrast, the predictive modeling of multiple psychosocial factors allows for the identification of constellations of risk and protection that may inform preventive strategies while also enabling interpretation at both the scale and item levels. For example, within the optimism domain, future-oriented positive expectations made the strongest contribution, highlighting specific cognitive dimensions underlying scale-level associations.^74^ Moreover, relying solely on direct questions about suicidal ideation may fail to identify individuals at risk because of stigma or nondisclosure, which are common in mental health contexts.^75^ In this context, predictive models based on broader psychosocial profiles might complement direct assessment by identifying vulnerability patterns even when suicidal ideation is not explicitly reported.

This study demonstrated the potential of machine learning models to predict suicidal ideation risk in the academic community and highlights factors beyond depressive symptoms (optimism, childhood emotional maltreatment, and loneliness). The findings highlight the importance of creating supportive academic settings that nurture a sense of belonging and encourage optimism about the future, thereby helping to protect mental health. Additionally, when prior experiences, such as childhood maltreatment, are considered, key vulnerability factors may be discovered, which could help prioritize psychological support initiatives within the academic community. We hope that this study’s insights contribute to the development of targeted interventions to enhance mental well-being in academic settings.

## Contributors

OFJ, LCLP, PMOF, DCMS, IAD, AVM, EV, FE, MGP, and LO conceptualized the study design. OFJ, PMOF, and AVM performed the formal analyses. OFJ, LCLP, PMOF, and AVM acquired the data and managed the resources. OFJ and LCLP were involved in data interpretation and drafted the original manuscript, preparing the figures and interpreting the data. DCMS and LO secured funding. All the authors contributed to manuscript revision and approved the final version. LO and MGP contributed equally to the senior authorship of this work. All the authors accessed and verified the data. All authors were responsible for the decision to submit the manuscript.

## Data sharing statement

The data underlying this study cannot be made publicly available because of ethical concerns regarding participant confidentiality and restrictions imposed by the ethics committee. Researchers interested in accessing the data can submit a request to the corresponding author. Requests will be evaluated on the basis of scientific merit, and access may be granted following the approval of a research proposal and, where applicable, a signed data access agreement. Deidentified individual participant data underlying the results reported in this article, along with a data dictionary, may be shared under these conditions. Data will be available upon publication. Additional documents, including the study protocol and statistical analysis plan, will also be available upon request.

## Use of artificial intelligence

Generative AI was used solely to improve the English readability (grammar, spelling, and style) of sections of this manuscript. All scientific content, analyses, and conclusions were generated by the authors. No AI tools were used to produce scientific insights, analyze or interpret data, or draw conclusions. The AI-assisted edits were performed under human oversight, and all the text was reviewed and approved by the authors. ChatGPT 5.2 (OpenAI) was used to suggest language edits. Example prompts included “improving the clarity of writing and English language without changing meaning” and “condensing this text to 250 words, preserving technical accuracy”. All suggestions were selectively incorporated and fully verified by the authors. The English language of the final manuscript was also reviewed by American Journal Experts (AJE). The authors take full responsibility for the content of the published article.

## Declaration of interests

The authors declare that they have no competing interests. The research was conducted in the absence of any commercial or financial relationships that could be construed as potential conflicts of interest.
