# Supplementary material for: Predicting suicidal ideation in academic communities using machine learning methods: a cross-sectional study
Source: Lancet Reg Health Am. 2026 Apr 30;59:101487. doi: 10.1016/j.lana.2026.101487 (PMC13141476; doi:10.1016/j.lana.2026.101487)
Supplement: Supplementary materials [file mmc1.pdf]

SUPPLEMENTARY MATERIALS

Title: Predicting suicidal ideation in academic communities using machine learning methods: a cross-sectional study.

Tables of content:

Supplementary Method 2
- Study design and psychometric assessments 2
- Machine learning classification models 2
  - Algorithms 3
  - Cross-validation strategies and hyperparameters optimization 3
  - Performance metrics 3
  - Models interpretation - weights 3
- Technical overview of multiple kernel learning (MKL) algorithm 4
- Additional analysis using synthetic minority oversampling technique (SMOTE) applied to the classification models 5
- Supplementary Table 1. CHERRIES for the PSIcovidA web-based survey: 7

Supplementary Results 10
- Supplementary Table 2. Demographic characteristics for the entire collected sample. 10
- Supplementary Table 3. Summary of performance metrics for all the algorithms and models tested. 11
- Supplementary Table 4. Detailed results for each iteration of the L1-multiple kernel learning model (5-fold cross-validation; hyperparameter values: 0.1, 1, 10, 1000). 12
- Supplementary Table 5. Detailed results for each iteration of the support vector machine model (5-fold cross-validation; hyperparameter values: 0.1, 1, 10, 1000). 13
- Supplementary Table 6. Detailed results for each iteration of the L2-logistic regression model (5-fold cross-validation; hyperparameter values: 0.1, 1, 10, 1000). 14
- Supplementary Table 7. Detailed results for all the kernel weights and item weights for the L1-MKL, SVM and L2-LR classification with fifty random combination models. 15
- Supplementary Table 8. Model performance metrics for the original and SMOTE-augmented data in RStudio. 16
- Supplementary Table 9. Model performance metrics for the original and SMOTE-augmented data in PRoNTo. 16

Supplementary Figure 1. Weights for the decision functions of the support vector machine and L2-regularized logistic regression models. 17

References: 18

Supplementary Method

Study design and psychometric assessments

The survey platform collected participants' email addresses as part of its default configuration, and this information was used solely to identify and remove duplicate responses; no additional personal identifiers were collected. Responses were downloaded from Google Forms to the restricted-access computers of the research team for analysis, without direct identifiers, in compliance with Brazilian ethical regulations. The participants accessed the survey (Google Forms) and provided voluntary informed consent.

Depressive symptom was assessed using the PHQ-9 items 1–8,[1,2] excluding item 9 (suicide ideation), and each item was entered as a separate feature in the model. The following items were used to assess symptom frequency over two weeks: (1) anhedonia, (2) depressed mood, (3) sleep disturbances, (4) fatigue or energy loss, (5) appetite or weight changes, (6) guilt or worthlessness, (7) concentration difficulties, and (8) psychomotor agitation or retardation. The internal consistency assessed by Cronbach's alpha was 0.920. Each item has a score ranging from 0 ("not once") to 3 ("almost every day").

The Three-Item Loneliness Scale (TILS) was developed and validated by Hughes and colleagues to assess individuals' perceptions of loneliness.[3,4] Items ask "How often do you feel…" with the following response options: (1) "hardly ever", (2) "some of the time", and (3) "often". Higher scores indicate greater levels of loneliness. The internal consistency as assessed by Cronbach's alpha in the present study was 0.857.

Emotional abuse and emotional neglect were assessed with the Portuguese adapted version of the Childhood Trauma Questionnaire (CTQ).[5,6] The CTQ includes five items for each subtype of childhood maltreatment (physical, emotional, and sexual abuse) as well as physical and emotional neglect. Only the emotional subtypes of childhood maltreatment (emotional abuse and emotional neglect) were used. This selection yields 10 items, plus 3 minimization and denial items. The participants responded on a 5-point Likert scale (1 = "never" to 5 = "always"), indicating the frequency of such experiences. Items related to emotional neglect are reverse-scored and must be coded inversely during construct computation. Childhood maltreatment was incorporated into the classification models as two distinct subcategories. The internal consistency as assessed by Cronbach's alpha was 0.918 for the whole scale.

The Life Orientation Test-Revised (LOT-R) is a questionnaire designed to assess the way individuals perceive their lives, either in a more or a less optimistic manner.[7,8] The LOT-R is a 10-item questionnaire consisting of a series of statements to which individuals respond on the basis of their agreement or disagreement. Three positive, three negative, and four filler items (excluded from the analyses) were rated from 0 ("strongly disagree") to 4 ("strongly agree"). Negative items (3, 7, 9) are reverse-scored and were inverted to calculate the total score. This measure reflects an individual's tendency to expect positive outcomes and maintain a positive perspective in various life situations. The internal consistency in the present study, as assessed by Cronbach's alpha, was 0.823 for the whole scale.

Machine learning classification models

The dataset included psychometric and sociodemographic variables, along with labels indicating individuals with and without suicidal ideation (SI). Analyses were conducted using the Pattern Recognition for Neuroimaging Toolbox (PRoNTo, version 3; Schrouff et al.[9]). During the training phase, the pattern recognition algorithm receives labeled training patterns and learns a predictive function that is subsequently applied in the test phase to predict labels (e.g., with or without SI)

for new psychometric patterns. All the data were normalized and mean-centered following the standard procedures of the PRoNTo.

Algorithms

In addition to the multiple-kernel learning (MKL) algorithm, support vector machines (SVMs)[10] and L2-regularized logistic regression (L2-logistic regression)[11] were implemented to test the robustness of the classification models. The SVM objective is to determine an optimal hyperplane that separates classes by maximizing the margin defined by the support vectors; when the data are not linearly separable, kernel functions project inputs into a higher-dimensional space in which linear separation becomes feasible.[10,12] L2-LR is a linear, nonkernel approach that operates directly in the original feature space, with the ℓ2 penalty constraining coefficient magnitudes to mitigate overfitting, enhance stability, and improve generalization.[11,13] With respect to preprocessing, all the features were mean-centered on the training dataset, and all the psychometric variables were z-scored for the non-kernel algorithms.

Cross-validation strategies and hyperparameters optimization

With respect to cross-validation strategies and the handling of class imbalance, the procedures of random selection, fold assignment, training, and testing were repeated fifty times, and we report the means, standard deviations, confidence intervals, and minimum and maximum performance values. Nested fivefold cross-validation comprised an internal loop for hyperparameter optimization and an external loop for performance assessment. Alternative k-fold strategies ($k = 2$ and $k = 10$) were also examined using PRoNTo's subsample option, which randomly selects subjects from the largest classes to match with the smallest group sample. We explored several hyperparameter grids—4 values [0.1, 1, 10, 1,000]; 6 [0.01; 0.1; 1; 10; 100; 1000]; and 3 [0.001; 1; 1000]—and, owing to the computational cost of permutation testing, we ultimately adopted the 4-value grid intervals.

Performance metrics

For performance evaluation, the metrics used were the balanced accuracy, class-specific accuracy, receiver-operating characteristic curves with area under the curve (AUC), precision, recall, specificity, and F1 scores. The class-specific accuracy, defined as the proportion correctly classified within each group (with and without SI); receiver-operating characteristic (ROC) curves, which summarize the trade-off between sensitivity and the false positive rate across decision thresholds; and the area under the curve (AUC), which provides an overall index of discrimination, with 0.5 indicating chance-level performance and 1.0 indicating perfect classification. Precision represents the proportion of correctly identified positives among all the predicted positives, recall (sensitivity) is the proportion of true positives correctly detected, specificity is the proportion of true negatives correctly classified, and the F1 score is the harmonic mean of precision and recall. The aggregated metrics across the fifty resampled models are presented in Table 2 and Figure 3.

Models interpretation - weights

For model interpretation, we quantified the contribution of both entire psychometric scales and individual items to the linear predictive function, which can be explicitly computed and visualized. Multiple-kernel algorithms produce two sets of weights: kernel weights, reflecting the overall contribution of each psychometric scale, and item weights, representing the influence of each specific question. Because each cross-validation fold generates a distinct weight vector, the final psychometric weight is obtained by averaging across folds and normalizing it by its Euclidean norm. Positive weights indicate stronger contributions toward identifying individuals in the SI group (higher risk of SI), whereas negative weights indicate stronger contributions

toward identifying individuals in the group without SI (lower risk of SI). The kernel weights and item-level contributions from the psychometric and demographic variables across the 50 classification models with random subsampling are summarized in Figure 4.

Technical overview of multiple kernel learning (MKL) algorithm

The MKL was originally proposed by Rakotomamonjy and colleagues[14] and was later adapted for the PRoNTo framework by Schrouff et al.[15] In kernel methods, the predictor is expressed as a weighted combination of kernel functions. For SVM-like models, the learned function has the following general form:

$$f(x) = \sum_{i=1}^{n} \alpha_i \; K(x, x_i) + b$$

See *Equation 1* in Rakotomamonjy et al.[14]

In this formulation, it $f(x)$ represents the decision function for a new input x, n denotes the number of training samples, α_i are the learned weights (Lagrange multipliers) associated with each training sample x_i, $K(x, x_i)$ computes the similarity between the new input and the training data in the feature space, and b is the bias term of the decision boundary.

Rather than using a single kernel, MKL replaces a single kernel with a weighted combination of M base kernels:

$$K(x, x') = \sum_{m=1}^{M} d_m \; K_m(x, x'), d_m \geq 0, \sum_{m=1}^{M} d_m = 1,$$

Here, each kernel $K_m(x, x')$ corresponds to a specific psychometric instrument or demographic variable (feature set), and the weights d_m are learned coefficients that indicate each kernel's contribution to the final combination. The constraints $d_m \geq 0$ and $\sum_{(m=1)}^{M} \quad d_m = 1$ ensure that the weights form a convex combination. In our setting, the learned weights d_m provide a direct measure of instrument-level contribution.

Simple MKL is derived from a convex optimization problem where the decision function is decomposed into components f_m living in different reproducing kernel Hilbert spaces (RKHSs) or feature spaces, each associated with a kernel k_m. The primal optimization problem includes a weighted regularization term and l_1-type constraint on kernel weights that encourages sparse kernel combinations (Rakotomamonjy et al., [14] *Equation 2*) as follows:

$$min_{\{f_m\}, b, \xi, d} \; \frac{1}{2} \sum_{m=1}^{M} \frac{1}{d_m} \; \| \, f_m \, \|_{H_m}^2 + C \sum_{i=1}^{n} \xi_i$$

subject to

$$y_i \left(\sum_{m=1}^{M} f_m \, (x_i) + b \right) \geq 1 - \xi_i, \xi_i \geq 0, d_m \geq 0, \sum_{m=1}^{M} d_m = 1.$$

In this optimization formulation, it f_m represents the decision function associated with the m**-th** kernel in the Hilbert space H_m, and $\| \, f_m \, \|_{H_m}^2$ is the squared norm acting as a regularization term. The slack variables ξ_i allow margin violations for misclassified or near-boundary samples, whereas hyperparameter $\boldsymbol{C}$ controls the trade-off between margin maximization and error minimization. The constraint $y_i \big(\sum_{m=1}^{M} f_m \, (x_i) + b \big) \geq 1 - \xi_i$ ensures correct classification with

a margin of at least *1*. When, $d_m \geq 0$, the corresponding components are forced toward $f_m \to 0$, yielding kernel selection through sparsity at the kernel/feature level.

A key advantage of MKL is its interpretability: the learned weights d_m reveal which kernels (psychometric scales or feature subsets) contribute most to the final decision function. This hierarchical structure facilitates interpretation at the kernel level (psychometric scale) while allowing within-item weights (individual items or questions).[15,16]

Additional analysis using synthetic minority oversampling technique (SMOTE) applied to the classification models

Previous studies have reported modest and sometimes inconsistent gains in predictive performance when subsampling strategies are applied in suicide prediction models.[17,18] Nevertheless, subsampling approaches such as the synthetic minority oversampling technique (SMOTE) are typically employed in machine learning studies addressing class imbalance in suicidal ideation and suicide attempt prediction.[19,20]

To assess the robustness of the main findings to an alternative strategy for handling class imbalance, additional models were tested using a SMOTE-based approach. These supplementary analyses were designed as sensitivity analyses and were not intended to replicate the primary multiple kernel learning (MKL) model but rather to evaluate whether the observed patterns are sensitive to the choice of subsampling strategy when using widely adopted linear classifiers. The analyses were performed using RStudio and PRoNTo (v.3).

Initially, the original dataset in RStudio[21] was split into training and testing data, using *initial_split* from the *tidymodels* package,[22] stratifying by the SI column (80% for training and 20% for testing) to guarantee balanced labels in both sets. We tested two different datasets. In the first dataset, we fit a logistic regression (LR) and a support vector machine (SVM) model using the original dataset, without any subsampling, to balance the minority-majority labels. In the second dataset, we added an oversampling step: we applied SMOTE to the training data to balance the minority–majority labels.

Synthetic Minority Oversampling Technique for Nominal and Continuous (SMOTE-NC) is a classical oversampling approach described in more detail in Chawla, Bowyer, and Hall.[23] The main idea behind this technique is to create synthetic data from minority class examples using their nearest neighbors (K-nearest neighbor), randomly selecting one or more neighbors and generating a synthetic sample in the feature space between these points. The training data were used to tune the hyperparameters with a 5-fold cross-validation, stratifying by the SI column. For the ridge logistic regression,[24] the lambda (penalty) parameter was optimized via a grid search between a sequence from 10^{-5} to 10^{-2} (length out = 20). For the support vector machine[25] with a linear kernel, the grid search for the cost parameter was a sequence from 10^{3} to 10^{2} (length out = 20). The best model was selected to be used for the testing dataset. The model's performance metrics are shown in **Supplementary Table 8**.

The R-based analyses focused exclusively on LR and SVM, as these models have well-established and stable implementations within the R ecosystem. The primary MKL analyses were conducted exclusively in PRoNTo, which provides a validated framework specifically designed for multiple kernel learning.

In the second step, we implemented the same predefined training and testing splits in PRoNTo (v.3) to assess the methodological reproducibility across software implementations. The training (80%) and testing (20%) partitions generated in R were fixed and imported into PRoNTo for both datasets (original and SMOTE-augmented).

Accordingly, this procedure differs from the standard PRoNTo pipeline, which relies on repeated nested cross-validation. Here, the models were trained once on the predefined training set (80% of the dataset) and evaluated on the held-out test set (20%) rather than being repeatedly re-estimated across folds. Within the training set, the data were further partitioned into five folds for hyperparameter tuning, following standard procedures. The regularization parameter grid was fixed at [0.1, 1, 10, 1000]. This simplified strategy was adopted to ensure direct comparability with the R-based analyses and to evaluate the robustness of the findings under an alternative implementation. We tested the same algorithms: multiple kernel learning (MKL),[14] support vector machines (SVMs),[10] and L2-regularized logistic regression (L2-LR).[11] The model's performance metrics are shown in **Supplementary Table 9**.

Supplementary Table 1. CHERRIES for the PSIcovidA web-based survey:

CHERRIES item[26]	Description in the PSIcovidA study
Design of the study	Cross-sectional analysis nested in a longitudinal cohort (PSIcovidA), conducted via an anonymous web-based survey targeting the Brazilian academic community during the return to in-person activities after the COVID-19 pandemic.
Institutional Review Board approval and informed consent	The study was approved by the Ethics Research Committee of Fluminense Federal University (UFF) and the Brazilian National Research Ethics Commission (CAAE: 52739721.0.0000.5243). Participants accessed the Google Forms survey link and were first presented with an electronic informed consent form. Only individuals who provided voluntary electronic consent could proceed to the questionnaires.
Data protection and privacy	Data were initially stored on Google Forms' secure servers. Participants were asked to provide their email addresses, which were used solely to identify and remove duplicate responses; no additional personal identifiers were intentionally collected. After data collection, responses were downloaded and stored on restricted-access computers by the research team, without direct identifiers, in compliance with Brazilian ethical regulations.
Development and testing	The survey was implemented on the Google Forms platform. The questionnaire was developed by a multidisciplinary team (psychologists, psychiatrists, and neuroscientists) based on previous instruments used by the PSIcovidA project and validated scales (e.g., PHQ-9, TILS, CTQ, LOT-R). The form was pilot tested internally to verify clarity, skip logic, average completion time, and technical functioning before field deployment.
Open versus closed survey	The survey was open and accessible online, with an unrestricted, shareable link. Participation was intended to be single per individual. To minimize multiple participation, we used email address collection (Google Forms default configuration) to identify and exclude duplicate responses.
Advertising the survey/contact mode	The survey link was disseminated via institutional channels (university and research institute mailing lists), personal academic networks, student and staff groups, online messaging platforms (e.g., WhatsApp), and the project's social media profile (@projetopsicovida). No face-to-face recruitment was conducted.
Context	Data collection took place in Brazil during the return to in-person academic activities following the acute phase of the COVID-19 pandemic. The survey focused on mental health, stressors, and psychosocial factors among members of the academic community (faculty, students, postdoctoral researchers, and administrative staff).
Mandatory or voluntary participation	Participation was voluntary. Invitations clearly stated that participation was optional, that individuals could discontinue the survey at any time before submission, and that decline would have no consequences for academic or professional status. No incentives or compensation were provided.
Incentives	No monetary, material, or academic incentives were offered for participation.
Time/Date	Data collection was conducted from March 10 to June 10, 2022.
Randomization of items or questionnaires	The order of the questionnaire sections and items was fixed. No randomization of item order or rotation of response options was implemented.
Adaptive questioning (skip logic)	Limited adaptive questioning (skip logic) was implemented. Participants who indicated that they were not part of the academic community or who reported being under 18 years old were automatically directed to the final page displaying mental health guidance and contact for psychological support, without completing the full questionnaire. All other participants who met eligibility criteria responded to all questionnaires uniformly, including the core instruments (PHQ-9, TILS, CTQ, LOT-R) and sociodemographic questions.
Number of items	The survey comprised a total of approximately 60–70 items distributed across multiple sections: (1) email address collection (1 item); (2) academic affiliation and eligibility screening (7 items, with conditional subitems for faculty and students); (3) demographic characteristics, including age; gender was self-reported (women/men); transgender identity was assessed

	separately through self-report and included categories such as cisgender, transgender, and nonbinary; race or ethnicity; state of residence; institution type; and caregiving responsibilities (approximately 15 items, with conditional branches); (4) pandemic-related experiences, including return to in-person activities, occupational exposure, vaccination status, and COVID-19 risk factors (approximately 10 items); (5) mental health history and changes during the pandemic (approximately 5 items, with conditional follow-ups); (6) the Patient Health Questionnaire-9 (PHQ-9; 9 items plus 1 functional impairment item) for depressive symptoms; (7) the Three-Item Loneliness Scale (TILS; 3 items); (8) the Childhood Trauma Questionnaire (CTQ); and (9) the Life Orientation Test – Revised (LOT-R; 10 items). The total number of items corresponded to an average completion time of approximately 25 minutes for eligible participants who completed the full questionnaire.
Number of screens (pages)	The questionnaire was presented over twenty (20) Google Forms screens: two (2) informed consent; five (5) sociodemographic and academic information; four (4) mental health history and pandemic-related experiences; three (3) parenthood and caregiving responsibilities; one (1) screen for each psychosocial scale (in total of 4); and mental health guidance and contact for psychological support (2).
Completeness check	In Google Forms, all psychometric questionnaire items were configured as mandatory (required fields), preventing participants from advancing without providing a response to each item. For sociodemographic questions, a "prefer not to answer" or "not declared" option was explicitly provided to allow participants to decline responding to sensitive items while still completing the survey. After data collection, responses categorized as "undeclared", including participants who selected "prefer not to answer" or "not declared," as well as those who selected "other" and provided open-text entries that could not be reliably classified, were excluded from analyses according to predefined criteria. Internal consistency for each psychometric scale (PHQ-9, TILS, CTQ, LOT-R) was evaluated and is reported in the Results section.
Review step	Participants could review and modify their responses within each page before advancing to the next section. Additionally, participants could return to previously completed pages to review and edit their responses at any time before final submission. After submission, responses could no longer be changed or accessed by participants.
Unique site visitor determination	Because this was a survey link disseminated via email and social media (not a public website with visitor tracking), we did not estimate the number of site visitors. Instead, we focused on the number of submitted survey responses.
Cookies used	No cookies were deliberately used by the research team for tracking participation. Google Forms' standard session management was employed, but no cookie data were accessed or stored by the investigators.
IP check	IP addresses were not collected or used for data cleaning or participant identification.
Log file analysis	Log file analysis was not performed. The only metrics recorded were timestamps of submission and email addresses (used solely for duplicate detection).
Registration	No prior registration or login was required to access the questionnaire. Participants accessed the survey directly via the shared link. Participants were asked to provide their email address as part of Google Forms' standard configuration, and this information was used solely for duplicate detection.
Handling of incomplete questionnaires	Partially completed questionnaires were automatically saved by Google Forms. For the present analyses, only respondents with complete psychometric data and classifiable sociodemographic information (gender identity, race or ethnicityy) were included. Responses categorized as "undeclared" (e.g., "prefer not to answer," "not declared," or "other" with unclassifiable text) were excluded according to predefined rules established prior to analysis.
Use of atypical timestamps	We did not establish explicit cutoffs based on completion time to exclude “atypical” responses. However, extremely short or obviously invalid entries were flagged during data cleaning and could be excluded if combined with other signs of poor data quality (e.g., duplicate email).
Statistical correction for nonrepresentative samples	The sample was a convenience, self-selected sample of the academic community and is not fully representative of the Brazilian academic population. No poststratification weights or other statistical adjustments for representativeness were applied. This limitation is acknowledged and discussed in the manuscript.

Sample size and flow of participants	A total of 4,326 responses were collected. Exclusion criteria were duplicate responses (n=42), nonacademic respondents (n=273), undeclared or misclassified nonbinary and transgender participants (n=40), undeclared racial/ethnic groups (n=105), undeclared information for both gender and race (n=11), and small subgroup sizes (nonbinary or transgender, n=25; Asian, n=34; Indigenous, n=10) limited their inclusion in statistical models, which may affect generalizability. The final analytic sample comprised 3,828 respondents (see Figure 1).
Recruitment channels and coverage	The survey link was disseminated via institutional channels (university and research institute mailing lists), personal academic networks, student and staff groups, and the project's social media profile (@projetopsicovida). This strategy mainly reached individuals affiliated with universities and research institutes (faculty, undergraduate and graduate students, postdoctoral researchers, administrative staff).
Reporting of response rate	Because the survey link was disseminated widely via email lists, messaging apps, and social media, the exact denominator (number of individuals who received or saw the invitation) was unknown, and a conventional response rate could not be calculated. We report the absolute number of submitted responses (N=4,326) and the final analytic sample (N=3,828).
Access to results	The main findings are reported in the present manuscript. Participants were informed that aggregate results might be disseminated in scientific articles, presentations, and on project communication channels. Individual-level feedback was not provided, but, upon completion of the survey, participants received general mental health guidance and contact information for psychological support services.

Supplementary Results

Supplementary Table 2. Demographic characteristics for the entire collected sample.

	Total sample N (%)	With SI N (%)	Without SI N (%)	Previous mental disorders N (%)	TILS Mean (95% CI)	CTQ-EA Mean (95% CI)	CTQ-EN Mean (95% CI)	LOT-R Mean (95% CI)	PHQ* Mean (95% CI)
Gender identity (self-reported)									
Cisgender men	1,302 (32.12)	216 (27.41)	1,086 (32.26)	376 (25.13)	5.04 (4.94-5.14)	8.89 (8.67-9.10)	10.76 (10.52-11.00)	15.27 (14.98-15.56)	7.00 (6.66-7.35)
Cisgender women	2,673 (65.95)	542 (68.78)	2,131 (65.27)	1,088 (72.73)	5.49 (5.42-5.56)	10.73 (10.54-10.91)	11.44 (11.26-11.62)	14.87 (14.66-15.08)	9.59 (9.34-9.85)
Transgender men	3 (0.07)	1 (0.13)	2 (0.06)	2 (0.13)	8.33 (7.03-9.64)	15.33 (11.05-19.62)	14.67 (10.38-18.95)	9.00 (-1.79-19.79)	13.33 (8.11-18.56)
Transgender women	2 (0.05)	2 (0.25)	0 (0.00)	2 (0.13)	6.00 (6.00-6.00)	13.00 (7.12-18.88)	13.00 (9.08-16.92)	13.50 (12.52-14.48)	17.00 (17.00-17.00)
Nonbinary	22 (0.54)	9 (1.14)	13 (0.40)	13 (0.87)	6.27 (5.44-7.11)	11.77 (9.64-13.91)	11.68 (9.19-14.18)	11.64 (9.07-14.21)	11.91 (9.12-14.70)
Not declared §	51 (1.26)	18 (2.28)	33 (1.01)	15 (1.00)	5.37 (4.90-5.85)	11.43 (10.13-12.74)	13.37 (12.02-14.72)	12.88 (11.31-14.46)	9.57 (7.88-11.26)
Race or ethnicity (self-reported)									
White individuals	2,598 (64.10)	428 (54.31)	2,170 (66.46)	950 (63.50)	5.24 (5.17-5.31)	9.84 (9.67-10.02)	11.02 (10.84-11.19)	15.21 (15.00-15.42)	8.31 (8.06-8.57)
Black individuals	1,294 (31.93)	318 (40.36)	976 (29.89)	488 (32.62)	5.55 (5.45-5.65)	10.70 (10.44-10.97)	11.66 (11.40-11.92)	14.52 (14.22-14.82)	9.73 (9.35-10.10)
Asian individuals	35 (0.86)	8 (1.02)	27 (0.83)	10 (0.67)	4.89 (4.26-5.51)	9.11 (7.62-10.55)	10.71 (9.12-12.30)	15.20 (13.59-16.81)	6.43 (4.62-8.24)
Indigenous individuals	10 (0.25)	2 (0.25)	8 (0.25)	6 (0.40)	5.50 (4.06-6.94)	12.10 (8.74-15.46)	11.60 (8.00-15.20)	14.00 (12.06-15.94)	10.40 (5.85-14.95)
Not declared §	116 (2.86)	32 (4.06)	84 51 (2.57)	42 (2.81)	5.58 (5.19-5.97)	11.11 (10.18-12.05)	12.03 (11.21-12.86)	13.87 (12.76-14.99)	9.28 (7.93-10.64)
Total	4,053 (100)	788 (100)	3,265 (100)	1,496 (100)					

Abbreviations: Data are presented as N(%), where percentages represent the proportion within each group and sum to 100% within each column. The columns represent the total sample (all participants), with SI sample (participants with suicidal ideation) and without SI sample (participants without suicidal ideation). Note: §Not declared includes participants who selected “prefer not to answer” or provided unclassifiable responses. Mean values and 95% CI: confidence interval mean (lower, upper); SI: suicidal ideation; TILS: Three-Item Loneliness Scale; CTQ: Childhood Trauma Questionnaire; EA: emotional abuse; EN: emotional neglect; LOT-R: Life Orientation Test-Revised; PHQ: depressive symptoms; *PHQ—assessed by the sum of items 1-8 depressive symptoms, excluding item 9.

Supplementary Table 3. Summary of performance metrics for all the algorithms and models tested.

Algorithms[a,b]	Balance Accuracy	Class: With SI	Class: Without SI	AUC	Precision	Specificity	Recall	F1-score
Classification models with fifty random subsamples groups (mean and 95% CI)								
MKL [5f p4]	77.61* (77.40-77.82)	76.30* (76.08-76.51)	78.93* (78.60-79.25)	0.86* (0.86-0.86)	0.78 (0.78-0.79)	0.79 (0.786-0.792)	0.76 (0.761-0.765)	0.77 (0.771-0.775)
MKL [5f p6]	77.61 (77.39-77.82)	76.28 (76.06-76.49)	78.93 (78.60-79.26)	0.86 (0.86-0.86)	0.78 (0.781-0.786)	0.79 (0.786-0.792)	0.76 (0.761-0.765)	0.773 (0.771-0.765)
SVM [5f p4]	78.23* (76.38-79.64)	77.07* (74.36-80.05)	79.39* (77.42-81.31)	0.86* (0.84-0.87)	0.789 (0.77-0.80)	0.79 (0.77-0.81)	0.77 (0.744-0.813)	0.780 (0.759-0.795)
L2-LR [5f p4]	78.57* (78.37-78.78)	77.92* (77.69-78.14)	79.23* (78.96-79.50)	0.87* (0.869-0.873)	0.79 (0.787-0.792)	0.79 (0.790-0.795)	0.78 (0.777-0.782)	0.784 (0.782-0.786)
Classification models using standard undersampling procedure								
MKL [10f p6]	79.36	79.21	79.51	0.88	0.79	0.79	0.79	0.79
MKL [2f p6]	78.05	76.87	79.22	0.87	0.79	0.79	0.77	0.78
MKL [5f p3]	79.22	79.07	79.37	0.88	0.79	0.79	0.79	0.79
MKL [5f p4]	78.87	78.38	79.37	0.87	0.79	0.79	0.78	0.79
MKL [5f p6]	78.94	79.38	79.51	0.87	0.79	0.79	0.78	0.79

Abbreviations: SI, suicidal ideation; mean values and 95% confidence interval (lower-upper); AUC, area under the curve of the receiver–operating characteristic; MKL, multiple kernel learning; SVM, support vector machine; L2-LR, L2-logistic regression. *The *p values* were obtained by a 100-permutation test ($p<0.05$). [a]cross-validation (k-folds): ***5f***: 5-fold, ***10f***: 10-fold, ***2f***: 2-fold. [b]Hyperparameter optimization: ***p4***: [0.1; 1; 10; 1000]; ***p6***: [0.01; 0.1; 1; 10; 100; 1000]; ***p3***: [0.001; 1; 1000]

Supplementary Table 4. Detailed results for each iteration of the L1-multiple kernel learning model (5-fold cross-validation; hyperparameter values: 0.1, 1, 10, 1000).

Random-undersampling	Balance Accuracy	*P values*	Class With SI	*P values*	Class Without SI	*P values*	AUC	*P values*
iteration_01	77.56	p = 0.0099	76.44	p = 0.0099	78.68	p = 0.0099	0.87	p = 0.0099
iteration_02	78.53	p = 0.0099	76.58	p = 0.0099	80.48	p = 0.0099	0.86	p = 0.0099
iteration_03	77.28	p = 0.0099	76.72	p = 0.0099	77.85	p = 0.0099	0.86	p = 0.0099
iteration_04	77.77	p = 0.0099	76.03	p = 0.0099	79.51	p = 0.0099	0.86	p = 0.0099
iteration_05	78.81	p = 0.0099	76.99	p = 0.0099	80.63	p = 0.0099	0.87	p = 0.0099
iteration_06	77.49	p = 0.0099	75.89	p = 0.0099	79.09	p = 0.0099	0.86	p = 0.0099
iteration_07	78.46	p = 0.0099	76.99	p = 0.0099	79.93	p = 0.0099	0.86	p = 0.0099
iteration_08	79.36	p = 0.0099	77.69	p = 0.0099	81.04	p = 0.0099	0.87	p = 0.0099
iteration_09	77.21	p = 0.0099	76.44	p = 0.0099	77.98	p = 0.0099	0.85	p = 0.0099
iteration_10	77.63	p = 0.0099	76.16	p = 0.0099	79.09	p = 0.0099	0.86	p = 0.0099
iteration_11	77.35	p = 0.0099	76.72	p = 0.0099	77.99	p = 0.0099	0.86	p = 0.0099
iteration_12	77.63	p = 0.0099	75.47	p = 0.0099	79.78	p = 0.0099	0.87	p = 0.0099
iteration_13	76.94	p = 0.0099	74.64	p = 0.0099	79.23	p = 0.0099	0.86	p = 0.0099
iteration_14	78.25	p = 0.0099	77.00	p = 0.0099	79.51	p = 0.0099	0.86	p = 0.0099
iteration_15	77.56	p = 0.0099	76.99	p = 0.0099	78.13	p = 0.0099	0.87	p = 0.0099
iteration_16	77.14	p = 0.0099	76.44	p = 0.0099	77.85	p = 0.0099	0.86	p = 0.0099
iteration_17	76.93	p = 0.0099	75.75	p = 0.0099	78.12	p = 0.0099	0.86	p = 0.0099
iteration_18	77.28	p = 0.0099	76.03	p = 0.0099	78.53	p = 0.0099	0.87	p = 0.0099
iteration_19	76.80	p = 0.0099	76.17	p = 0.0099	77.43	p = 0.0099	0.85	p = 0.0099
iteration_20	77.91	p = 0.0099	76.03	p = 0.0099	79.79	p = 0.0099	0.87	p = 0.0099
iteration_21	78.32	p = 0.0099	75.61	p = 0.0099	81.03	p = 0.0099	0.87	p = 0.0099
iteration_22	78.39	p = 0.0099	77.69	p = 0.0099	79.10	p = 0.0099	0.87	p = 0.0099
iteration_23	76.73	p = 0.0099	74.92	p = 0.0099	78.54	p = 0.0099	0.86	p = 0.0099
iteration_24	77.01	p = 0.0099	75.20	p = 0.0099	78.82	p = 0.0099	0.87	p = 0.0099
iteration_25	78.88	p = 0.0099	77.13	p = 0.0099	80.62	p = 0.0099	0.87	p = 0.0099
iteration_26	77.63	p = 0.0099	75.33	p = 0.0099	79.93	p = 0.0099	0.86	p = 0.0099
iteration_27	77.21	p = 0.0099	76.99	p = 0.0099	77.43	p = 0.0099	0.86	p = 0.0099
iteration_28	77.77	p = 0.0099	75.89	p = 0.0099	79.66	p = 0.0099	0.86	p = 0.0099
iteration_29	77.56	p = 0.0099	76.99	p = 0.0099	78.12	p = 0.0099	0.86	p = 0.0099
iteration_30	77.29	p = 0.0099	76.44	p = 0.0099	78.13	p = 0.0099	0.86	p = 0.0099
iteration_31	78.18	p = 0.0099	77.13	p = 0.0099	79.23	p = 0.0099	0.87	p = 0.0099
iteration_32	77.77	p = 0.0099	77.55	p = 0.0099	77.99	p = 0.0099	0.86	p = 0.0099
iteration_33	76.87	p = 0.0099	75.89	p = 0.0099	77.84	p = 0.0099	0.86	p = 0.0099
iteration_34	76.87	p = 0.0099	75.47	p = 0.0099	78.27	p = 0.0099	0.86	p = 0.0099
iteration_35	76.66	p = 0.0099	75.61	p = 0.0099	77.71	p = 0.0099	0.86	p = 0.0099
iteration_36	77.56	p = 0.0099	75.89	p = 0.0099	79.23	p = 0.0099	0.86	p = 0.0099
iteration_37	76.94	p = 0.0099	75.47	p = 0.0099	78.40	p = 0.0099	0.86	p = 0.0099
iteration_38	77.91	p = 0.0099	76.86	p = 0.0099	78.95	p = 0.0099	0.87	p = 0.0099
iteration_39	79.29	p = 0.0099	76.71	p = 0.0099	81.86	p = 0.0099	0.88	p = 0.0099
iteration_40	76.45	p = 0.0099	75.48	p = 0.0099	77.43	p = 0.0099	0.85	p = 0.0099
iteration_41	77.00	p = 0.0099	76.02	p = 0.0099	77.99	p = 0.0099	0.85	p = 0.0099
iteration_42	77.63	p = 0.0099	76.44	p = 0.0099	78.81	p = 0.0099	0.86	p = 0.0099
iteration_43	76.31	p = 0.0099	75.33	p = 0.0099	77.29	p = 0.0099	0.84	p = 0.0099
iteration_44	77.01	p = 0.0099	76.30	p = 0.0099	77.72	p = 0.0099	0.86	p = 0.0099
iteration_45	77.98	p = 0.0099	76.02	p = 0.0099	79.93	p = 0.0099	0.87	p = 0.0099
iteration_46	76.24	p = 0.0099	75.33	p = 0.0099	77.16	p = 0.0099	0.85	p = 0.0099
iteration_47	78.81	p = 0.0099	77.13	p = 0.0099	80.49	p = 0.0099	0.87	p = 0.0099
iteration_48	78.11	p = 0.0099	75.89	p = 0.0099	80.34	p = 0.0099	0.87	p = 0.0099
iteration_49	77.70	p = 0.0099	77.41	p = 0.0099	77.98	p = 0.0099	0.86	p = 0.0099
iteration_50	78.67	p = 0.0099	77.55	p = 0.0099	79.78	p = 0.0099	0.87	p = 0.0099

Abbreviations: SI, suicidal ideation; AUC, area under the curve of the receiver–operating characteristic; The *P values* were obtained by a 100-permutation test ($p<0.05$).

Supplementary Table 5. Detailed results for each iteration of the support vector machine model (5-fold cross-validation; hyperparameter values: 0.1, 1, 10, 1000).

Random-undersampling	Balance Accuracy	*P values*	Class: With SI	*P value*	Class: Without SI	*P values*	AUC	*P values*
iteration_01	78.33	p = 0.0099	77.00	p = 0.0099	79.65	p = 0.0099	0.86	p = 0.0099
iteration_02	79.02	p = 0.0099	78.24	p = 0.0099	79.79	p = 0.0099	0.86	p = 0.0099
iteration_03	78.67	p = 0.0099	77.97	p = 0.0099	79.37	p = 0.0099	0.86	p = 0.0099
iteration_04	78.74	p = 0.0099	77.83	p = 0.0099	79.65	p = 0.0099	0.86	p = 0.0099
iteration_05	78.95	p = 0.0099	77.83	p = 0.0099	80.08	p = 0.0099	0.86	p = 0.0099
iteration_06	77.28	p = 0.0099	77.14	p = 0.0099	77.42	p = 0.0099	0.85	p = 0.0099
iteration_07	78.39	p = 0.0099	77.00	p = 0.0099	79.79	p = 0.0099	0.86	p = 0.0099
iteration_08	78.67	p = 0.0099	77.00	p = 0.0099	80.35	p = 0.0099	0.86	p = 0.0099
iteration_09	78.18	p = 0.0099	76.72	p = 0.0099	79.65	p = 0.0099	0.85	p = 0.0099
iteration_10	78.33	p = 0.0099	77.28	p = 0.0099	79.37	p = 0.0099	0.85	p = 0.0099
iteration_11	78.60	p = 0.0099	77.14	p = 0.0099	80.06	p = 0.0099	0.86	p = 0.0099
iteration_12	78.60	p = 0.0099	76.86	p = 0.0099	80.34	p = 0.0099	0.87	p = 0.0099
iteration_13	77.70	p = 0.0099	75.61	p = 0.0099	79.79	p = 0.0099	0.85	p = 0.0099
iteration_14	78.19	p = 0.0099	77.00	p = 0.0099	79.37	p = 0.0099	0.85	p = 0.0099
iteration_15	79.16	p = 0.0099	80.05	p = 0.0099	78.27	p = 0.0099	0.86	p = 0.0099
iteration_16	77.01	p = 0.0099	76.45	p = 0.0099	77.57	p = 0.0099	0.86	p = 0.0099
iteration_17	77.00	p = 0.0099	76.03	p = 0.0099	77.98	p = 0.0099	0.85	p = 0.0099
iteration_18	78.53	p = 0.0099	77.14	p = 0.0099	79.92	p = 0.0099	0.87	p = 0.0099
iteration_19	77.56	p = 0.0099	76.58	p = 0.0099	78.54	p = 0.0099	0.85	p = 0.0099
iteration_20	78.88	p = 0.0099	77.14	p = 0.0099	80.62	p = 0.0099	0.86	p = 0.0099
iteration_21	78.87	p = 0.0099	76.44	p = 0.0099	81.31	p = 0.0099	0.86	p = 0.0099
iteration_22	79.23	p = 0.0099	78.80	p = 0.0099	79.66	p = 0.0099	0.87	p = 0.0099
iteration_23	76.38	p = 0.0099	74.36	p = 0.0099	78.40	p = 0.0099	0.84	p = 0.0099
iteration_24	79.29	p = 0.0099	77.55	p = 0.0099	81.03	p = 0.0099	0.87	p = 0.0099
iteration_25	78.67	p = 0.0099	77.00	p = 0.0099	80.34	p = 0.0099	0.86	p = 0.0099
iteration_26	78.12	p = 0.0099	76.86	p = 0.0099	79.38	p = 0.0099	0.85	p = 0.0099
iteration_27	77.84	p = 0.0099	76.86	p = 0.0099	78.82	p = 0.0099	0.85	p = 0.0099
iteration_28	78.53	p = 0.0099	77.00	p = 0.0099	80.07	p = 0.0099	0.86	p = 0.0099
iteration_29	77.77	p = 0.0099	76.86	p = 0.0099	78.68	p = 0.0099	0.85	p = 0.0099
iteration_30	78.46	p = 0.0099	78.25	p = 0.0099	78.68	p = 0.0099	0.85	p = 0.0099
iteration_31	78.88	p = 0.0099	78.11	p = 0.0099	79.65	p = 0.0099	0.86	p = 0.0099
iteration_32	78.67	p = 0.0099	77.97	p = 0.0099	79.37	p = 0.0099	0.86	p = 0.0099
iteration_33	77.70	p = 0.0099	75.75	p = 0.0099	79.65	p = 0.0099	0.85	p = 0.0099
iteration_34	77.63	p = 0.0099	75.75	p = 0.0099	79.51	p = 0.0099	0.85	p = 0.0099
iteration_35	78.19	p = 0.0099	75.61	p = 0.0099	80.76	p = 0.0099	0.86	p = 0.0099
iteration_36	78.32	p = 0.0099	76.72	p = 0.0099	79.93	p = 0.0099	0.86	p = 0.0099
iteration_37	77.63	p = 0.0099	76.16	p = 0.0099	79.10	p = 0.0099	0.85	p = 0.0099
iteration_38	79.09	p = 0.0099	77.69	p = 0.0099	80.48	p = 0.0099	0.86	p = 0.0099
iteration_39	79.64	p = 0.0099	78.80	p = 0.0099	80.48	p = 0.0099	0.86	p = 0.0099
iteration_40	77.56	p = 0.0099	75.89	p = 0.0099	79.24	p = 0.0099	0.85	p = 0.0099
iteration_41	77.84	p = 0.0099	77.55	p = 0.0099	78.12	p = 0.0099	0.85	p = 0.0099
iteration_42	76.80	p = 0.0099	76.17	p = 0.0099	77.42	p = 0.0099	0.84	p = 0.0099
iteration_43	77.42	p = 0.0099	77.14	p = 0.0099	77.71	p = 0.0099	0.84	p = 0.0099
iteration_44	77.29	p = 0.0099	76.59	p = 0.0099	77.99	p = 0.0099	0.85	p = 0.0099
iteration_45	79.15	p = 0.0099	77.69	p = 0.0099	80.61	p = 0.0099	0.87	p = 0.0099
iteration_46	77.22	p = 0.0099	75.75	p = 0.0099	78.68	p = 0.0099	0.85	p = 0.0099
iteration_47	79.36	p = 0.0099	77.96	p = 0.0099	80.76	p = 0.0099	0.87	p = 0.0099
iteration_48	77.49	p = 0.0099	77.27	p = 0.0099	77.72	p = 0.0099	0.85	p = 0.0099
iteration_49	78.46	p = 0.0099	77.69	p = 0.0099	79.23	p = 0.0099	0.86	p = 0.0099
iteration_50	78.18	p = 0.0099	77.14	p = 0.0099	79.23	p = 0.0099	0.86	p = 0.0099

Abbreviations: SI, suicidal ideation; AUC, area under the curve of the receiver–operating characteristic; The *P values* were obtained by a 100-permutation test ($p<0.05$).

Supplementary Table 6. Detailed results for each iteration of the L2-logistic regression model (5-fold cross-validation; hyperparameter values: 0.1, 1, 10, 1000).

Random-undersampling	Balance Accuracy	*P values*	Class With SI	P values	Class Without SI	*P values*	AUC	*P values*
iteration_01	79.02	p = 0.0099	78.25	p = 0.0099	79.79	p = 0.0099	0.87	p = 0.0099
iteration_02	79.01	p = 0.0099	78.11	p = 0.0099	79.92	p = 0.0099	0.88	p = 0.0099
iteration_03	78.74	p = 0.0099	78.25	p = 0.0099	79.23	p = 0.0099	0.87	p = 0.0099
iteration_04	79.08	p = 0.0099	78.24	p = 0.0099	79.92	p = 0.0099	0.87	p = 0.0099
iteration_05	79.51	p = 0.0099	78.24	p = 0.0099	80.77	p = 0.0099	0.88	p = 0.0099
iteration_06	78.94	p = 0.0099	78.38	p = 0.0099	79.50	p = 0.0099	0.87	p = 0.0099
iteration_07	78.25	p = 0.0099	78.11	p = 0.0099	78.40	p = 0.0099	0.87	p = 0.0099
iteration_08	79.15	p = 0.0099	77.41	p = 0.0099	80.89	p = 0.0099	0.88	p = 0.0099
iteration_09	77.77	p = 0.0099	76.72	p = 0.0099	78.82	p = 0.0099	0.87	p = 0.0099
iteration_10	77.84	p = 0.0099	77.28	p = 0.0099	78.40	p = 0.0099	0.87	p = 0.0099
iteration_11	79.01	p = 0.0099	78.24	p = 0.0099	79.78	p = 0.0099	0.87	p = 0.0099
iteration_12	79.29	p = 0.0099	77.55	p = 0.0099	81.03	p = 0.0099	0.88	p = 0.0099
iteration_13	78.74	p = 0.0099	78.25	p = 0.0099	79.23	p = 0.0099	0.87	p = 0.0099
iteration_14	78.46	p = 0.0099	77.56	p = 0.0099	79.37	p = 0.0099	0.87	p = 0.0099
iteration_15	79.23	p = 0.0099	79.77	p = 0.0099	78.68	p = 0.0099	0.88	p = 0.0099
iteration_16	77.84	p = 0.0099	77.97	p = 0.0099	77.71	p = 0.0099	0.87	p = 0.0099
iteration_17	77.28	p = 0.0099	76.17	p = 0.0099	78.39	p = 0.0099	0.87	p = 0.0099
iteration_18	78.87	p = 0.0099	78.11	p = 0.0099	79.64	p = 0.0099	0.88	p = 0.0099
iteration_19	78.05	p = 0.0099	78.25	p = 0.0099	77.85	p = 0.0099	0.86	p = 0.0099
iteration_20	79.01	p = 0.0099	78.52	p = 0.0099	79.51	p = 0.0099	0.87	p = 0.0099
iteration_21	79.22	p = 0.0099	77.56	p = 0.0099	80.89	p = 0.0099	0.88	p = 0.0099
iteration_22	79.16	p = 0.0099	79.08	p = 0.0099	79.23	p = 0.0099	0.88	p = 0.0099
iteration_23	77.35	p = 0.0099	76.03	p = 0.0099	78.68	p = 0.0099	0.86	p = 0.0099
iteration_24	79.99	p = 0.0099	79.49	p = 0.0099	80.48	p = 0.0099	0.88	p = 0.0099
iteration_25	79.64	p = 0.0099	78.38	p = 0.0099	80.89	p = 0.0099	0.87	p = 0.0099
iteration_26	79.09	p = 0.0099	77.69	p = 0.0099	80.48	p = 0.0099	0.87	p = 0.0099
iteration_27	79.23	p = 0.0099	79.36	p = 0.0099	79.10	p = 0.0099	0.87	p = 0.0099
iteration_28	78.53	p = 0.0099	77.69	p = 0.0099	79.37	p = 0.0099	0.88	p = 0.0099
iteration_29	78.60	p = 0.0099	78.39	p = 0.0099	78.82	p = 0.0099	0.87	p = 0.0099
iteration_30	78.46	p = 0.0099	77.97	p = 0.0099	78.95	p = 0.0099	0.87	p = 0.0099
iteration_31	78.60	p = 0.0099	78.38	p = 0.0099	78.82	p = 0.0099	0.87	p = 0.0099
iteration_32	78.81	p = 0.0099	78.38	p = 0.0099	79.23	p = 0.0099	0.87	p = 0.0099
iteration_33	78.39	p = 0.0099	77.27	p = 0.0099	79.50	p = 0.0099	0.87	p = 0.0099
iteration_34	78.05	p = 0.0099	77.83	p = 0.0099	78.27	p = 0.0099	0.86	p = 0.0099
iteration_35	77.50	p = 0.0099	76.86	p = 0.0099	78.13	p = 0.0099	0.87	p = 0.0099
iteration_36	77.98	p = 0.0099	76.72	p = 0.0099	79.23	p = 0.0099	0.87	p = 0.0099
iteration_37	77.70	p = 0.0099	76.72	p = 0.0099	78.68	p = 0.0099	0.87	p = 0.0099
iteration_38	79.85	p = 0.0099	78.94	p = 0.0099	80.75	p = 0.0099	0.87	p = 0.0099
iteration_39	79.50	p = 0.0099	79.08	p = 0.0099	79.92	p = 0.0099	0.88	p = 0.0099
iteration_40	77.35	p = 0.0099	76.86	p = 0.0099	77.84	p = 0.0099	0.86	p = 0.0099
iteration_41	78.25	p = 0.0099	77.83	p = 0.0099	78.67	p = 0.0099	0.87	p = 0.0099
iteration_42	77.62	p = 0.0099	77.55	p = 0.0099	77.69	p = 0.0099	0.86	p = 0.0099
iteration_43	77.28	p = 0.0099	76.72	p = 0.0099	77.85	p = 0.0099	0.85	p = 0.0099
iteration_44	77.91	p = 0.0099	77.41	p = 0.0099	78.41	p = 0.0099	0.87	p = 0.0099
iteration_45	78.81	p = 0.0099	77.69	p = 0.0099	79.93	p = 0.0099	0.88	p = 0.0099
iteration_46	77.98	p = 0.0099	77.97	p = 0.0099	77.99	p = 0.0099	0.86	p = 0.0099
iteration_47	79.50	p = 0.0099	78.24	p = 0.0099	80.76	p = 0.0099	0.88	p = 0.0099
iteration_48	78.25	p = 0.0099	78.11	p = 0.0099	78.40	p = 0.0099	0.87	p = 0.0099
iteration_49	78.46	p = 0.0099	78.24	p = 0.0099	78.67	p = 0.0099	0.87	p = 0.0099
iteration_50	78.60	p = 0.0099	78.11	p = 0.0099	79.10	p = 0.0099	0.88	p = 0.0099

Abbreviations: SI, suicidal ideation; AUC, area under the curve of the receiver–operating characteristic; The *P values* were obtained by a 100-permutation test ($p<0.05$).

Supplementary Table 7. Detailed results for all the kernel weights and item weights for the L1-MKL, SVM and L2-LR classification with fifty random combination models.

	**L1-MKL*1**	**SVM*2**	**L2-LR*2**
	Mean % (95% CI: lower-upper)	Mean % (95% CI: lower-upper)	Mean % (95% CI: lower-upper)
**Depression symptoms - PHQ**	**46.83 (44.73-48.92)**	**39.08 (38.14-40.02)**	**40.29 (39.23-41.36)**
q.1 (anhedonia)	0.10 (0.08-0.12)	0.08 (0.06-0.10)	0.12 (0.11-0.14)
q.2 (depressed mood)	0.65 (0.63-0.66)	0.51 (0.49-0.52)	0.50 (0.48-0.51)
q.3 (sleep disturbances)	0.12 (0.10-0.13)	0.05 (0.04-0.07)	0.05 (0.04-0.07)
q.4 (fatigue or energy loss)	-0.13 (-0.15- -0.12)	-0.06 (-0.08- -0.05)	-0.07 (-0.09- -0.06)
q.5 (appetite or weight changes)	0.06 (0.04-0.07)	-0.02 (-0.03 - -0.002)	0.03 (0.01-0.05)
q.6 (guilt or worthlessness)	0.61 (0.60-0.62)	0.45 (0.43-0.46)	0.52 (0.51-0.53)
q.7 (concentration difficulties)	-0.08 (-0.10- -0.07)	-0.17 (-0.18- -0.15)	-0.11 (-0.13- -0.10)
q.8 (psychomotor aspects)	0.36 (0.35-0.38)	0.28 (0.27-0.30)	0.21 (0.20-0.23)
**Optimism – LOT-R**	**13.89 (13.49-14.30)**	**12.97 (12.23-13.70)**	**14.38 (13.61-15.16)**
q.01 (hope for best)	-0.054 (-0.10- -0.02)	0.03 (0.02-0.04)	0.03 (0.02-0.04)
q.03 (expect the worst)*	-0.148 (-0.19 - -0.11)	0.003 (-0.01-0.014)	-0.04 (-0.05- -0.02)
q.04 (optimism about the future)	-0.291 (-0.33- -0.25)	-0.06 (-0.07- -0.05)	-0.06 (-0.07- -0.05)
q.07 (anticipating disappointment)*	0.117 (0.07-0.16)	0.06 (0.04-0.07)	0.06 (0.05-0.07)
q.09 (rarely expect good things)*	-0.290 (-0.34 - -0.24)	-0.10 (-0.117- -0.09)	-0.08 (-0.10- -0.07)
q.10 (hope good things)	-0.830 (-0.85- -0.80)	-0.26 (-0.28- -0.24)	-0.28 (-0.30- -0.27)
**CTQ - Emotional Abuse**	**11.74 (11.16-12.32)**	**11.33 (10.67-11.98)**	**13.54 (12.75-14.33)**
q.03 (called me names)	0.14 (0.08-0.20)	0.01 (-0.004-0.02)	-0.004 (-0.02-0.01)
q.08 (unwanted child)	0.60 (0.55-0.66)	0.12 (0.11-0.13)	0.17 (0.16-0.19)
q.14 (hurtful words)	0.06 (0.004-0.12)	0.01 (-0.004-0.03)	0.02 (0.002-0.04)
q.18 (hated by family)	-0.23 (-0.29- -0.18)	-0.13 (-0.14- -0.11)	-0.12 (-0.14- -0.11)
q.25 (emotionally abused)	0.60 (0.55-0.66)	0.15 (0.14-0.17)	0.17 (0.16-0.19)
**CTQ - Emotional Neglect**	**10.79 (10.11-11.467)**	**13.89 (12.91-14.88)**	**14.26 (13.28-15.24)**
q.05 (made me feel special)*	-0.13 (-0.19- -0.08)	-0.12 (-0.14- -0.10)	-0.05 (-0.07- -0.04)
q.07 (felt loved)*	0.70 (0.65-0.75)	0.24 (0.22-0.25)	0.23 (0.21-0.24)
q.13 (family took care of each other)*	0.53 (0.47-0.58)	0.09 (0.07-0.11)	0.13 (0.12-0.15)
q.19 (family felt united)*	-0.10 (-0.164- -0.04)	-0.07 (-0.09- -0.06)	-0.08 (-0.09- -0.06)
q.28 (family source of support)*	-0.05 (-0.11-0.01)	0.04 (0.03-0.06)	-0.06 (-0.08- -0.04)
**Loneliness – TILS**	**9.43 (8.72-10.15)**	**6.89 (6.31-7.47)**	**5.62 (5.16- 6.09)**
q.1 (felt lack of companionship)	0.66 (0.58-0.75)	0.02 (0.01-0.04)	0.05 (0.04-0.07)
q.2 (felt left out)	0.34 (0.23-0.44)	0.16 (0.14-0.19)	0.13 (0.11-0.15)
q.3 (felt isolated)	0.27 (0.16-0.38)	0.04 (0.02-0.06)	-0.002 (-0.02-0.01)
**Demographic**	**7.31 (6.72-7.91)**	**15.84 (15.01-16.67)**	**11.90 (11.26-12.53)**
Women	0.001 (-0.08-0.09)	-0.06 (-0.06- -0.04)	-0.04 (-0.05- -0.03)
Men	-0.001 (-0.07-0.08)	0.05 (0.04-0.06)	0.04 (0.03-0.05)
White individuals	-0.13 (-0.20- -0.05)	-0.12 (-0.13- -0.11)	-0.08 (-0.09- -0.08)
Black individuals	0.13 (0.05-0.20)	0.12 (0.11-0.13)	0.08 (0.08-0.09)
No previous mental disorder	-0.51 (-0.57- -0.45)	-0.13 (-0.15- -0.13)	-0.07 (-0.07- -0.06)
Previous mental disorder	0.51 (0.45-0.57)	0.14 (0.13-0.15)	0.07 (0.06-0.07)
Age	-0.20 (-0.24- -0.16)	-0.04 (-0.04- -0.03)	-0.11 (-0.13- -0.10)

Abbreviations: CI: confidence intervals of the mean, PHQ: Patient Health Questionnaire, LOT-R: Life Orientation Test-Revised, CTQ: Childhood Trauma Questionnaire, TILS: Three-Item Loneliness Scale. *1Multiple kernel learning (MKL) model: weights are represented as *kernel weights* (scale level) and *item weights* (question level). *2SVM and L2LR models: item weights represent the direct weight vectors, averaged across fifty random models with confidence intervals derived from the distribution. Scale-level feature importance was calculated by summing the absolute values of the item weights within each scale and then expressing this sum as a percentage of the total absolute weight across all features. This percentage quantifies the relative contribution of each scale to the model's predictive function. *Items were reverse scored to ensure directional consistency within each construct, with higher scores uniformly representing greater construct severity (e.g., lower optimism or higher neglect).

Supplementary Table 8. Model performance metrics for the original and SMOTE-augmented data in RStudio.

Dataset	No subsampling		SMOTE-NC	
**Algorithms/Metrics**	**L2-LR**	**SVM**	**L2-LR**	**SVM**
Precision	0.66	0.65	0.47	0.47
Recall	0.45	0.42	0.78	0.78
Specificity	0.94	0.95	0.80	0.80
F1-score	0.53	0.51	0.59	0.59
Accuracy	0.85	0.85	0.79	0.79
AUC	0.88	0.88	0.87	0.87

Abbreviations: L2-LR, L2-logistic regression; SVM, support vector machine; SMOTE-NC, synthetic minority oversampling technique for nominal and continuous features; AUC, area under curve.

Supplementary Table 9. Model performance metrics for the original and SMOTE-augmented data in PRoNTo.

Dataset	No subsampling			SMOTE		
**Algorithms/Metrics**	**L1-MKL**	**SVM**	**L2-LR**	**L1-MKL**	**SVM**	**L2-LR**
Balance Accuracy (%)	70.10	63.90	66.04	72.56	67.57	71.50
Class with SI (%)	47.59	33.10	39.31	89.66	84.83	82.07
Class without SI (%)	92.60	94.69	92.77	55.47	50.32	60.93
AUC	0.87	0.85	0.86	0.79	0.77	0.79
Precision	0.60	0.59	0.56	0.32	0.28	0.33
Specificity	0.93	0.95	0.93	0.55	0.50	0.65
Recall	0.48	0.33	0.39	0.90	0.85	0.82
F1-score	0.53	0.42	0.46	0.47	0.43	0.47

Abbreviations: SI, suicidal ideation; L1-MKL, multiple kernel learning; SVM, support vector machine; L2-LR, L2-logistic regression; SMOTE, synthetic minority oversampling technique; AUC, area under curve.

Supplementary Figure 1. Weights for the decision functions of the support vector machine and L2-regularized logistic regression models.

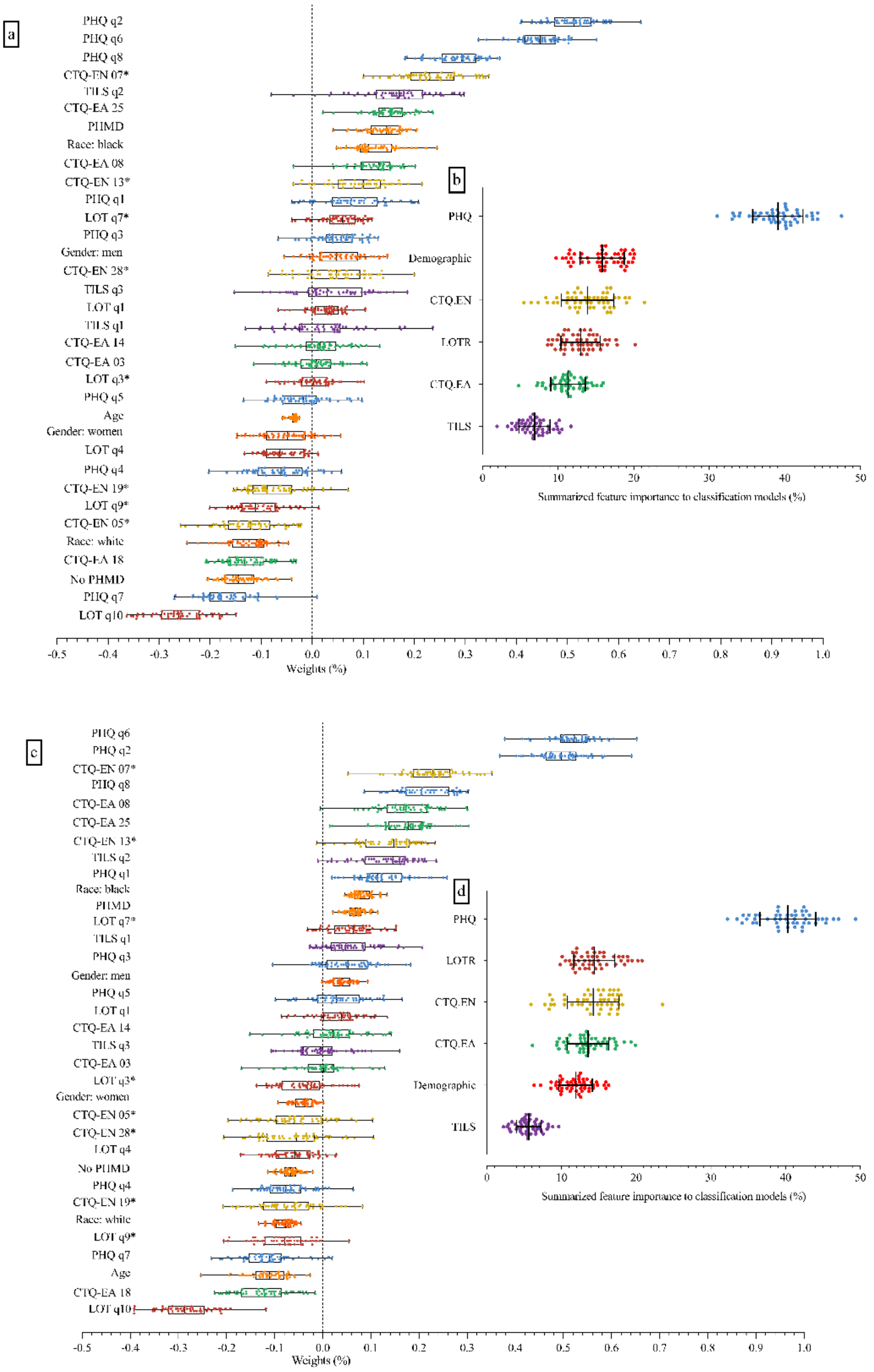

Legend: (**a**) Support vector machine weights for individual items across each psychometric scale. (**b**) Summarized SVM feature importance, expressed as the percentage contribution of each scale to the predictive function, based on the sum of absolute weights. (**c**) L2-regularized logistic regression weights for individual items across each psychometric scale. (**d**) Summarized L2LR feature importance, expressed as the percentage contribution of each scale to the predictive function, based on the sum of absolute weights. Box plots show the mean values and standard deviations (boxes), with whiskers indicating the minimum and maximum values. Each color represents a distinct psychometric scale or variable: PHQ: Patient Health Questionnaire (blue); LOT-R: Life Orientation Test-Revised (red); CTQ: Childhood Trauma Questionnaire; EA: emotional abuse (green); EN: emotional neglect (yellow); TILS: Three-Item Loneliness Scale (purple); demographic variables (orange); PHMD: previous history

of mental disorder. Each point represents the results from one of fifty random combination models. *Items were reverse scored to ensure directional consistency within each construct, with higher scores uniformly representing greater construct severity (e.g., lower optimism or higher neglect).

References:

1 Kroenke K, Spitzer RL, Williams JBW. The PHQ-9: Validity of a brief depression severity measure. *J Gen Intern Med* 2001; **16**: 606–13.

2 Santos IS, Tavares BF, Munhoz TN, *et al.* Sensibilidade e especificidade do Patient Health Questionnaire-9 (PHQ-9) entre adultos da população geral. *Cad Saúde Pública* 2013; **29**: 1533–43.

3 Hughes ME, Waite LJ, Hawkley LC, Cacioppo JT. A Short Scale for Measuring Loneliness in Large Surveys: Results From Two Population-Based Studies. *Res Aging* 2004; **26**: 655–72.

4 Russell D, Peplau LA, Cutrona CE. The revised UCLA Loneliness Scale: Concurrent and discriminant validity evidence. *Journal of Personality and Social Psychology* 1980; **39**: 472–80.

5 Bernstein DP, Stein JA, Newcomb MD, *et al.* Development and validation of a brief screening version of the Childhood Trauma Questionnaire. *Child Abuse & Neglect* 2003; **27**: 169–90.

6 Grassi-Oliveira R, Stein LM, Pezzi JC. Tradução e validação de conteúdo da versão em português do Childhood Trauma Questionnaire. *Rev Saúde Pública* 2006; **40**: 249–55.

7 Bandeira M, Bekou V, Lott KS, Teixeira MA, Rocha SS. Validação transcultural do teste de orientação da vida (TOV-R). *Estud psicol (Natal)* 2002; **7**: 251–8.

8 Scheier MF, Carver CS, Bridges MW. Distinguishing optimism from neuroticism (and trait anxiety, self-mastery, and self-esteem): A reevaluation of the Life Orientation Test. *Journal of Personality and Social Psychology* 1994; **67**: 1063–78.

9 Schrouff J, Rosa MJ, Rondina JM, *et al.* PRoNTo: Pattern Recognition for Neuroimaging Toolbox. *Neuroinform* 2013; **11**: 319–37.

10 Cortes C, Vapnik V. Support-vector networks. *Mach Learn* 1995; **20**: 273–97.

11 Ng AY. Feature selection, L_1 vs. L_2 regularization, and rotational invariance. In: Twenty-first international conference on Machine learning - ICML '04. Banff, Alberta, Canada: ACM Press, 2004: 78.

12 Cervantes J, Garcia-Lamont F, Rodríguez-Mazahua L, Lopez A. A comprehensive survey on support vector machine classification: Applications, challenges and trends. *Neurocomputing* 2020; **408**: 189–215.

13 Qin J, Lou Y. L_{1-2} Regularized Logistic Regression. In: 2019 53rd Asilomar Conference on Signals, Systems, and Computers. Pacific Grove, CA, USA: IEEE, 2019: 779–83.

14 Rakotomamonjy A, Francis B, Canu S, Grandvalet Y. SimpleMKL. *Journal of Machine Learning Research* 2008; : 2491–521.

15 Schrouff J, Mourão-Miranda J, Phillips C, Parvizi J. Decoding intracranial EEG data with multiple kernel learning method. *Journal of Neuroscience Methods* 2016; **261**: 19–28.

16 Fernandes Jr O, Portugal LCL, Alves RDCS, *et al.* Decoding negative affect personality trait from patterns of brain activation to threat stimuli. *NeuroImage* 2017; **145**: 337–45.

17 Pigoni A, Delvecchio G, Turtulici N, *et al.* Machine learning and the prediction of suicide in psychiatric populations: a systematic review. *Transl Psychiatry* 2024; **14**: 1–22.

18 Somé NH, Noormohammadpour P, Lange S. The use of machine learning on administrative and survey data to predict suicidal thoughts and behaviors: a systematic review. *Front Psychiatry* 2024; **15**. DOI:10.3389/fpsyt.2024.1291362.

19 Li Y, Yang Y, Song P, Duan L, Ren R. An improved SMOTE algorithm for enhanced imbalanced data classification by expanding sample generation space. *Sci Rep* 2025; **15**: 23521.

20 Parghi N, Chennapragada L, Barzilay S, *et al.* Assessing the predictive ability of the Suicide Crisis Inventory for near-term suicidal behavior using machine learning approaches. *Int J Methods Psych Res* 2021; **30**: e1863.

21 R Core Team. R: A language and environment for statistical computing. 2025. https://www.R-project.org/.

22 Max Kuhn and Hadley Wickham. Tidymodels: a collection of packages for modeling and machine learning using tidyverse principles. 2020. https://www.tidymodels.org.

23 Chawla NV, Bowyer KW, Hall LO, Kegelmeyer WP. SMOTE: Synthetic Minority Over-sampling Technique. *jair* 2002; **16**: 321–57.

24 Friedman J, Hastie T, Tibshirani R, *et al.* glmnet: Lasso and Elastic-Net Regularized Generalized Linear Models. 2008; : 4.1-10.

25 Karatzoglou A, Smola A, Hornik K. kernlab: Kernel-Based Machine Learning Lab. 2004; : 0.9-33.

26 Eysenbach G. Improving the Quality of Web Surveys: The Checklist for Reporting Results of Internet E-Surveys (CHERRIES). *J Med Internet Res* 2004; **6**: e34.
